# Signed weighted gene co-expression network analysis of transcriptional regulation in murine embryonic stem cells

**DOI:** 10.1186/1471-2164-10-327

**Published:** 2009-07-20

**Authors:** Mike J Mason, Guoping Fan, Kathrin Plath, Qing Zhou, Steve Horvath

**Affiliations:** 1 Statistics, University of California, Los Angeles, CA, 90095, USA; 2 Human Genetics, David Geffen School of Medicine, Los Angeles, CA, 90095, USA; 3 Biological Chemistry, University of California, Los Angeles, CA, 90095, USA; 4 Biostatistics, School of Public Health, University of California, Los Angeles, CA, 90095, USA

## Abstract

**Background:**

Recent work has revealed that a core group of transcription factors (TFs) regulates the key characteristics of embryonic stem (ES) cells: pluripotency and self-renewal. Current efforts focus on identifying genes that play important roles in maintaining pluripotency and self-renewal in ES cells and aim to understand the interactions among these genes. To that end, we investigated the use of unsigned and signed network analysis to identify pluripotency and differentiation related genes.

**Results:**

We show that signed networks provide a better systems level understanding of the regulatory mechanisms of ES cells than unsigned networks, using two independent murine ES cell expression data sets. Specifically, using signed weighted gene co-expression network analysis (WGCNA), we found a pluripotency module and a differentiation module, which are not identified in unsigned networks. We confirmed the importance of these modules by incorporating genome-wide TF binding data for key ES cell regulators. Interestingly, we find that the pluripotency module is enriched with genes related to DNA damage repair and mitochondrial function in addition to transcriptional regulation. Using a connectivity measure of module membership, we not only identify known regulators of ES cells but also show that Mrpl15, Msh6, Nrf1, Nup133, Ppif, Rbpj, Sh3gl2, and Zfp39, among other genes, have important roles in maintaining ES cell pluripotency and self-renewal. We also report highly significant relationships between module membership and epigenetic modifications (histone modifications and promoter CpG methylation status), which are known to play a role in controlling gene expression during ES cell self-renewal and differentiation.

**Conclusion:**

Our systems biologic re-analysis of gene expression, transcription factor binding, epigenetic and gene ontology data provides a novel integrative view of ES cell biology.

## Background

Embryonic stem (ES) cells have two important characteristics: pluripotency, the ability to differentiate into any type of cell in the body, and self-renewal, the ability to replicate indefinitely. As such, they have tremendous therapeutic potential for regenerative medicine [1,2]. Current work focuses on understanding and extending the network of genes that controls these key characteristics [3-16]. These efforts identified ES cell-specific transcription factors (TFs) that are differentially expressed between ES cells and differentiated cells (fibroblasts). Several studies have identified the targets of these TFs and the mechanism by which they regulate them [4,8,17]. Highly differentially expressed TFs (Oct4, Sox2, c-Myc, and Klf4) have been found capable of reprogramming fibroblasts to a pluripotent state [3].

While standard differential expression analysis techniques have led to remarkable discoveries they ignore the strong correlations that may exist between gene expression profiles. As a consequence, the user of a standard marginal analysis can drown in information but starve in knowledge. This is especially true when considering ES cells where many genes change expression during differentiation. For example, in a data set from Zhou *et al *2007, which we consider below, more than 6200 genes were highly differentially expressed (Student t-test p-value smaller than the very stringent threshold of 10^-6^). It is difficult to further prioritize these genes and to learn the underlying biological pathways. In contrast, co-expression networks, also referred to as 'association,' 'correlation,' or 'influence' networks [18-22], realize that genes can be highly correlated and thus can be grouped into large clusters (co-expression modules). For example, our network analysis of the same data organizes the genes into only 8 large modules. Next our module-centric analysis focuses on understanding the modules and their key regulators. Since it applies significance testing to the level of modules, co-expression network analysis may greatly alleviate the multiple testing problem that plagues standard gene-centric methods [23]. Gene co-expression network methods have been successfully applied in a variety of different settings [18,19,21,22,24-32].

In this article, we demonstrate that a co-expression network analysis of stem cell data sets provides novel biological insights that cannot be found using conventional techniques. Using external data (including gene ontology, TF binding data, epigenetic regulators), we also contrast the performance of signed and unsigned network construction methods. We find that signed co-expression network analysis performs best in this stem cell application. We identify pluripotency and differentiation related co-expression modules and novel ES cell regulators.

## Results and discussion

### Constructing Signed Co-expression Networks

We first define a gene co-expression similarity measure which is used to define the network. We denote the gene co-expression similarity measure of a pair of genes *i *and *j *by *s*_*ij*_. Many co-expression studies use the absolute value of the Pearson correlation as an unsigned co-expression similarity measure,

(1)

where gene expression profiles *x*_*i *_and *x*_*j *_consist of the expression of genes *i *and *j *across multiple microarray samples. However, using the absolute value of the correlation may obfuscate biologically relevant information, since no distinction is made between gene repression and activation. In contrast, in signed networks the similarity between genes reflects the sign of the correlation of their expression profiles. To define a signed co-expression measure between gene expression profiles *x*_*i *_and *x*_*j*_, we use a simple transformation of the correlation:

(2)

As the unsigned measure , the signed similarity  takes on a value between 0 and 1. Note that the unsigned similarity between two oppositely expressed genes (*cor*(*x*_*i*_, *x*_*j*_) = -1) equals 1 while it equals 0 for the signed similarity. Similarly, while the unsigned co-expression measure of two genes with zero correlation remains zero, the signed similarity equals 0.5.

Next, an adjacency matrix (network), *A *= [*a*_*ij*_], is used to quantify how strongly genes are connected to one another. *A *is defined by thresholding the co-expression similarity matrix *S *= [*s*_*ij*_]. 'Hard' thresholding (dichotomizing) the similarity measure *S *results in an unweighted gene co-expression network. Specifically an unweighted network adjacency is defined to be 1 if *s*_*ij *_> *τ *and 0 otherwise, i.e. two genes are considered connected if their similarity measure is above a given threshold *τ*, and are considered separated otherwise.

**Figure 1:**
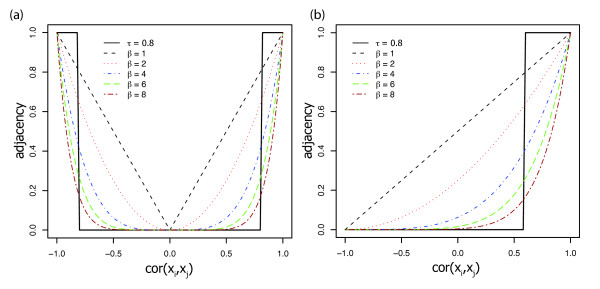
**Network Connection Strength Versus Expression Correlation**. Network adjacency (y-axis) versus correlation (x-axis) for an unweighted network (black step function with *τ *= 0.8) and weighted networks (dashed lines corresponding to different powers, *β*) in an unsigned network (**a**) and a signed network (**b**). Note that *cor*(*x*_*i*_, *x*_*j*_) = -1 leads to adjacency = 0 in the signed network. The weighted network preserves the continuous nature of the co-expression information while an unweighted network dichotomizes the correlation.

Because hard thresholding encodes gene connections in a binary fashion, it can be sensitive to the choice of the threshold and result in the loss of co-expression information [19]. The continuous nature of the co-expression information can be preserved by employing soft thresholding, which results in a weighted network. Specifically, we use a continuous measure to assess their connection strength:

(3)

where the power *β *is the thresholding parameter. As a default we use *β *= 6 and *β *= 12 for unsigned and signed networks, respectively. Alternatively, *β *and be chosen using the scale-free topology criterion [19]. Since *log*(*a*_*ij*_) = *β *× *log*(*s*_*ij*_), the weighted network adjacency is linearly related to the co-expression similarity on a logarithmic scale. Figure 1 shows the resulting adjacencies after applying the co-expression similarity measures and thresholding. Note that a high power *β *transforms high similarities into high adjacencies, while pushing low similarities towards 0. Since this soft-thresholding procedure leads to weighted adjacency matrix, the ensuing analysis is referred to as weighted gene co-expression network analysis or WGCNA [19,23,33,34].

A major step in our module centric analysis is to cluster genes into network modules using a network proximity measure. Roughly speaking, a pair of genes has a high proximity if it is closely interconnected. We will use the convention that the maximal proximity between two genes is 1 and the minimum proximity is 0. Specifically, we define the proximity as the topological overlap measure (TOM) [35-37] which can also be defined for weighted networks [19]. The TOM combines the adjacency of two genes and the connection strengths these two genes share with other "third party" genes (see equation 6 in the Methods section and Additional File 1). The TOM is a highly robust measure of network interconnectedness (proximity). This proximity is used as input of average linkage hierarchical clustering. Modules are defined as branches of the resulting cluster tree [38]. This module detection procedure has been used in many applications [23,25-30,32,39,40] and a comparison to alternative procedures is beyond the scope of this article.

We find it convenient to summarize the gene expression profiles of a given module with the module eigengene, which can be considered as the best summary of the standardized module expression data [33,41]. The module eigengene of a given module is defined as the first principal component of the standardized expression profiles (see equation 8 in the Methods section).

### Quantifying Module Membership

To identify possible regulators within a given module, we looked for highly connected intramodular hub genes, i.e. genes that have strong connections within the module. In our effort to find these genes, we examined two types of connectivity measures, which can be applied relative to any module *q*. The first connectivity measure is intramodular connectivity  defined as

(4)

where *n*^(*q*) ^is the number of genes in the *q*^*th *^module. In the case of an unweighted network,  simply counts the number of connections to gene *i *within the *q*^*th *^module. Intramodular connectivity can be interpreted as a measure of module membership: the higher the intramodular connectivity, the more centrally located the gene is in the module and the more certain is its membership with regard to this module. In signed networks, these highly connected hub genes may up-regulate adjacent genes since they are positively correlated with them, while in unsigned networks they may activate or repress their neighboring genes.

The second connectivity measure is the module eigengene based connectivity,  (also known as the signed module membership measure [33]), defined as

(5)

where *E*^(*q*) ^is the eigengene of the *q*^*th *^module (see equations 9 and 10 in the Methods section) and *x*_*i *_is the expression profile of the gene *i*. We denote modules by colors. For example,  denotes the module membership measure of the i-th gene with regard to the blue module.

Module eigengene based connectivity has several advantages over intramodular connectivity: first, it is naturally scaled to take on values between -1 and 1; second, one can use a correlation test to calculate a corresponding p-value for a gene's module membership; third it can be used in signed networks to identify genes that are anti-correlated with a given module eigengene (i.e. they may repress genes in the module), and fourth, *k*_*ME *_can be computed for any gene on the array (not just genes used in the network construction). In practice, we found that intramodular and module eigengene based connectivity are highly correlated (Additional File 2). A priori, the connectivity measures defined in equations 4 and 5 are quite different. But we show in the Methods section that a simple theoretical relationship between them can be derived in the context of a signed co-expression module. Due to its advantages, we used the module eigengene based connectivity  as the measure of module membership in our applications.

### Signed WGCNA Identifies Pluripotency Related Modules in Ivanova et al (2006) Data Set

We generated unsigned and signed co-expression networks to analyze over 17,000 genes measured across 70 expression arrays from data published in Ivanova *et al *(2006) [4]. This data set contains expression profiles of ES cells individually depleted for the transcription factors Oct4, Nanog, Sox2, Esrrb, and Tbx3 by RNA interference (RNAi). The data set also includes expression profiles for RNAi knock downs of Tcl1 a co-activator of AKT kinase, and an EST (Mm343880), along with expression profiles of control ES cells carrying an empty RNAi vector and of ES cells differentiated by retinoic acid (RA). Each of these treatments was sampled over approximately eight days. To compare the performances of unsigned and signed WGCNA in identifying gene groups that are important for the regulation of the pluripotent state, we defined gene modules in unsigned and signed networks and assessed module function and importance by determining gene ontology terms associated with each module and examining module membership of genes known to play a role in ES cells. In addition, we analyzed how genes of a given module are bound by chromatin regulators or pluripotency TFs by incorporating independent promoter binding information.

**Figure 2:**
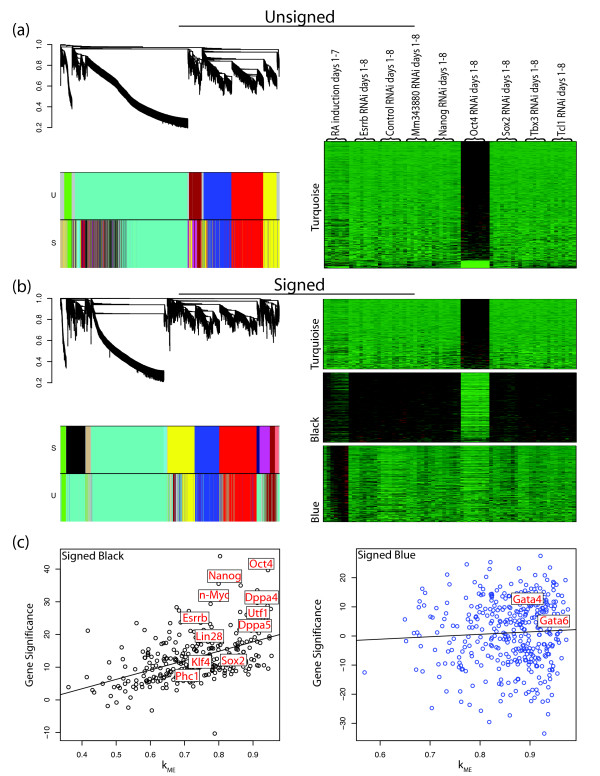
**Unsigned and Signed Mouse ES cell Networks in Ivanova et al**. **a, left**, Dendrogram of the unsigned network of the Ivanova *et al *(2006) data set with color bands below indicating module membership for the unsigned network (U) and the signed network (S). **a, right**, heat map for visualizing standardized gene expressions (rows) across samples (columns) for genes in the turquoise module in the unsigned network. **b, left**, Dendrogram of the signed network of the Ivanova *et al *(2006) data set with color bands below indicating module membership for the signed network (S) and the unsigned network (U). **b, right**, heat map of expression profiles across samples for genes in the turquoise, black, and blue modules in the signed network. Note, modules are not scaled to reflect the number of genes in each module. **c**, scatter plot of module membership, *k*_*ME*_, (x-axis) plotted against gene significance, GS, (y-axis) for the black and blue modules in the signed network with known ES cell regulators and differentiation genes labelled.

Figure 2a shows the dendrogram of the unsigned network for the Ivanova *et al *data set. Modules were found by cutting branches of the cluster tree (dendrogram), using the dynamic tree cut library in R [38]. Modules are indicated by the color bands below the dendrogram. Genes that do not clearly belong to a branch are colored grey. To compare modules in signed and unsigned networks, we show two color bands: the top color band shows the genes colored by module membership in the unsigned network (corresponding to the dendrogram), while the bottom color band shows genes colored by module membership in the signed network. Similarly, Figure 2b displays the dendrogram of the signed network with the top color band showing genes colored by module membership in the signed network and the bottom color band showing genes colored by module membership in the unsigned network. These figures show that while some large modules (turquoise, yellow, red, and blue) are preserved in both networks, the signed network has two distinct small modules (black and tan) hidden within the unsigned turquoise module. The black and tan modules from the signed network are scattered throughout the unsigned network's turquoise module and cannot be detected since there is no branch of the dendrogram corresponding directly to these modules (i.e. regardless of the tree cutting algorithm employed, these modules would not be found in the unsigned network, data not shown). Figure 2a also shows a heatmap of the expression profiles of genes in the turquoise module from the unsigned network. Genes in this module exhibit a positive (red) or negative (green) change in expression upon knock down of the master regulator Oct4, which is not surprising given that Oct4 RNAi has be shown to cause a distinct differentiation pattern from other TF RNAi's [4,42]. Heat maps are similarly shown in Figure 2b for the turquoise, black, and blue modules from the signed network. In contrast to the unsigned network, each module contains genes with similar expression profiles. Here the turquoise module is made of genes that are activated when Oct4 is knocked down, while the black module contains genes that are repressed when Oct4 is knocked down and are down-regulated during retinoic acid (RA) induced differentiation. The blue module contains genes that are activated upon differentiation. This analysis shows that signed WGCNA identifies modules with more specific expression patterns than unsigned WGCNA.

### Functional Enrichment with Regard to Known ES Cell Related Genes

Next we used external data to further study the gene modules defined by the networks and reveal their functional roles. We used two different strategies for this evaluation: first we assigned transcription factors and other regulators with known roles in pluripotency, self-renewal or differentiation to modules [4,8] and second, we incorporated genome-wide binding data for transcription factors and other regulators implicated in ES cell regulation in order to determine if these modules contain genes that are directly controlled by ES cell related TFs or differentiation suppressors [5,6,16].

Many genes known to maintain the pluripotent state of ES cells are found in the black module in the signed network. We defined a measure of gene significance (GS) as the t-statistic from the paired Student's t-test of expression in control RNAi samples and ES cell samples with RNAi knock down of Oct4 (paired by day of treatment). Figure 2c shows GS plotted against its module eigengene based connectivity, *k*_*ME*_, in the black and blue modules of the signed network with marker genes labeled. Since the signed module membership *k*_*ME *_is defined as the correlation between a gene expression profile and the module eigengene, its values lie between -1 and 1 with values near 1 signifying strong module membership to the corresponding signed module. Figure 2c shows a strong linear relationship between *k*_*ME *_and GS in the black module (correlation = 0.5, p-value = 6.5e-13). As expected, most of the genes whose RNAi knock down induced ES cell differentiation in Ivanova *et al *[4] belong to the black module (Oct4, Nanog, Sox2, Esrrb, and Dppa4, Fisher's exact test p-value = 3.2 × 10^-5^). Oct4's high connectivity ( = 0.94) makes it a hub gene in the black module, consistent with its known role as a master regulator of the pluripotent state. Furthermore, many genes that are known to be highly expressed in ES cells are also in the black module (e.g. Klf4, Utf1, and Phc1). Klf4 is one of the four TFs that can reprogram differentiated cells into a pluripotency state [3]. Utf1 interacts with Oct4, affects chromatin regulation in ES cells, and has recently been shown to improve reprogramming efficiency [43-45]. Phc1 is a Polycomb Group (PcG) protein. PcG proteins repress genes that become active upon differentiation of ES cells by mediating histone H3 lysine 27 tri-methylation and histone H2a ubiquitination [6]. The blue module contains Gata6 and Gata4, which are both highly connected ( = 0.93 and 0.88, respectively). These TFs are markers of ES cell differentiation, particularly into endoderm. Below we provide further evidence that the black and blue modules are related to pluripotency and differentiation respectively.

### Module Enrichment with Regard to Known ES Cell Regulators

We incorporated genome-wide binding data for TFs (Oct4, Sox2, Nanog, Stat3, Smad1, cMyc, nMyc, Zfx, E2f1) and other regulators (Suz12) implicated in the maintenance of pluripotency and self-renewal, which were obtained by chromatin immunoprecipitation (ChIP) and massive parallel sequencing (ChIP-seq) by Chen *et al *(2008) [16]. Oct4, Sox2, Nanog, Smad1, and Stat3 are referred to as the Oct4 group of TFs, as they have been shown to often co-bind genomic regions; cMyc, nMyc, E2f1, and Zfx are referred to as the cMyc group of TFs because they also co-bind genomic regions [16]. Together TFs in the Oct4 and cMyc group are thought to activate expression of genes involved in pluripotency and self-renewal. Suz12, is a subunit of the histone H3K27 methyltransferase PcG protein complex, which represses genes that are activated upon differentiation [6,16].

To determine if binding by the two TF groups and Suz12 occurs more often in certain modules we computed binding enrichment for each module. Enrichment is defined as the odds ratio, that is the probability of a gene being bound by a particular TF or TF complex for genes in a given module divided by the probability of being bound for genes not located in the module. Table 1 shows module enrichment of genes bound by TFs in the Oct4 group, the cMyc group, and Suz12 for modules in the unsigned and signed networks. A gene is called bound by the Oct4 or cMyc groups if it is bound by at least 4 of the 5 and 3 of the 4 TFs in each TF group, respectively (similar results are found when using 3 of 5 and 2 of 4, data not shown). In agreement with the notion that the black module found in the signed network contains genes that are implicated in pluripotency, this module is strongly enriched with genes bound by TFs in the Oct4 and cMyc groups and under-enriched for binding by Suz12. Specifically, the proportion of genes bound by the Oct4 group in the black module is almost twice the proportion of genes bound in the general population (1.94). Similarly genes in the black module are almost twice as likely to be bound by TFs in the cMyc group (1.87) and are almost three times less likely to be bound by Suz12 (0.344). These enrichments further support the idea that the black module is a pluripotency and self-renewal module. Other modules, like the blue, brown, and cyan, are enriched for Suz12 bound genes and under-enriched for genes bound by the two TF groups. The blue module is the most significantly enriched for Suz12 binding further supporting the idea that genes in this module are involved in ES cell differentiation. For modules preserved in both unsigned and signed networks, Table 1 shows that enrichment values are generally more significant in the signed network. Similar enrichments can be seen for Oct4 and Nanog binding from Loh *et al *(2006) and Polycomb Group (PcG) protein binding from Boyer *et al *(2006) (Additional File 3). The incorporation of binding data suggests that signed WGCNA better separates genes into modules based on function and regulation. Specifically, the black module can be considered a pluripotency/self-renewal module, while the blue, brown, and cyan modules can be considered differentiation modules in the signed network. It is important to reiterate that the pluripotency/self-renewal black module was only found using signed WGCNA.

**Table 1:** Transcription Factor Binding in Ivanova et al Networks

**Unsigned Network**				
**Module**	**No. of genes**	**Oct4Complex**	**cMycComplex**	**Suz12**
**blue**	899	0.863, 0.232	1.09, 0.0398	0.956, 0.366
**brown**	1241	0.862, 0.179	0.757, 3.09e-09	1.92, 2.5e-21
**green**	1556	0.825, 0.0832	0.811, 1.78e-07	1.13, 0.0496
**grey**	7407	1.16, 2.37e-05	1.18, 4.62e-45	0.866, 2.17e-07
**red**	1673	1.1, 0.169	1.68, 6.75e-74	0.281, 7.61e-28
**turquoise**	5721	0.898, 0.0268	0.801, 4.3e-36	1.2, 6.96e-10
**yellow**	1053	1.14, 0.15	1.46, 5.74e-22	0.458, 7.39e-10

**Signed Network**				
**black**	941	2.9, 7.25e-24	1.83, 4.8e-58	0.288, 1.95e-15
**blue**	220	0.608, 0.165	0.488, 1.59e-07	1.93, 4.42e-05
**brown**	1090	0.736, 0.0402	0.754, 2.04e-08	1.99, 3.27e-21
**cyan**	581	0.783, 0.174	0.931, 0.15	1.78, 2.58e-08
**green**	1538	0.731, 0.0138	0.778, 1.14e-09	1.12, 0.0534
**grey**	4428	0.949, 0.224	0.853, 2.38e-14	1.13, 0.000604
**magenta**	514	1.09, 0.286	1.11, 0.0548	0.824, 0.122
**midnightblue**	649	1.61, 0.00131	1.89, 2.87e-45	0.181, 6.99e-15
**pink**	2242	1.4, 4.41e-05	1.85, 8.63e-160	0.341, 3.32e-31
**red**	1270	1.01, 0.429	1.56, 1.19e-37	0.371, 8.07e-16
**salmon**	650	1.03, 0.388	1.19, 0.00083	0.67, 0.00378
**tan**	445	2.16, 4.62e-06	1.71, 4.65e-21	0.37, 3.14e-06
**turquoise**	5205	0.679, 2.41e-09	0.749, 3.51e-49	1.24, 2.71e-11
**yellow**	1042	1.05, 0.326	1.35, 1.9e-13	0.633, 4.69e-05

Enrichment for binding by the Oct4 and cMyc TF Groups and Suz12.

Each enrichment score (before the comma) is followed by its corresponding p-value (after the comma) calculated from the hyper-geometric distribution. P-values are uncorrected for multiple comparisons.

### Epigenetic Regulation and Module Membership

**Figure 3:**
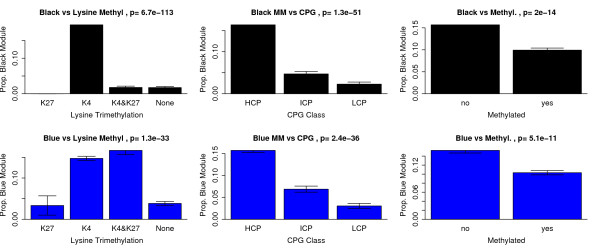
**Relating Module Membership to Epigenetic Regulation**. The top 1000 genes with highest module membership in the black module (top row) and blue module (bottom row) are related to 3 epigenetic variables (corresponding to the 3 columns). The y-axis reports the proportion of top 1000 genes that are known to belong to the group of genes defined on the x-axis. Histone H3K4me3 trimethylation status is abbreviated K4, H3K27me3 trimethylation statys is abbreviated by K27. Promoters that are both H3K4 and H3K27 trimethilated in ES cells (denoted *K*4&*K*27) are thought to poise key developmental genes for activation upon differentiation [50,51]. Note that genes with promoter CpG methylation are significantly (*p *= 2.0 × 10^-14^) under-enriched with respect to the top 1000 black module genes.

Recent studies suggest that chromatin structure and epigenetic modifications, like histone modification and DNA methylation, play a role in controlling gene expression during ES cell self-renewal and differentiation [46-51]. For example, gene repression by the PcG protein complex via histone H3 lysine 27 trimethylation (H3K27me3) is required for ES cell self-renewal and pluripotency [6,52]. To understand how epigenetic variables contribute to the regulation of ES cells we studied the relationship of the pluripotency and differentiation modules with ES cell H3K4 and H3K27 trimethylation, DNA methylation, and CpG promoter content from previously published data sets [50,51]. We related the epigenetic variables to module membership in the black (Figure 3, top row) and blue module (Figure 3, bottom row). Specifically, we determined what proportion of genes with a given epigenetic mark (H3K4me3 for example) are also in the top 1000 genes with the highest  (or ). Below we show that our findings are highly robust with respect to the number of selected module genes (see also Additional File 4). H3K4me3 is associated with gene activation, whereas H3K27me3 is known to silence genes. Figure 3 shows that genes with H3K4 trimethylation in ES cells contain significantly more black module genes than genes without this marker (Kruskal Wallis test p-value = 6.7 × 10^-113^) which may reflect the active role the pluripotency module plays in ES cells. Interestingly, genes with H3K4 trimethylation or bivalent methylation contain significantly (*p *= 1.3 × 10^-33^) more blue module genes than other gene classifications. Promoters that are both H3K4 and H3K27 trimethilated in ES cells (referred to as bivalent promoters) are thought to poise key developmental genes for activation upon differentiation [50].

Mammalian gene promoters are known to fall into one of at least two major classes: 1) CpG-rich promoters are associated with both ubiquitously expressed 'housekeeping' genes, and genes with more complex expression patterns, particularly those expressed during embryonic development and 2) CpG-poor promoters are generally associated with highly tissue-specific genes. To understand the role of CpG content in our modules we analyzed three CpG content classifications from Mikkelsen *et al*(2007): high (denoted HCP), low (LCP), and intermediate (ICP). Figure 3 shows that HCP genes contain significantly more black module genes (*p *= 1.3 × 10^-51^) and significantly more blue module genes (*p *= 2.4 × 10^-36^) than ICP or LCP genes. The LCPs are known to have a very different trimethylation pattern than the HCPs. Few (6.5%) of LCPs have significant H3K4me3 in ES cells and virtually none have H3K27me3. HCPs and LCPs are subject to distinct modes of regulation. In ES cells, all HCPs seem to be targets of trithorax group activity, and may therefore drive transcription unless actively repressed by PcG proteins. In contrast, LCPs seem to be inactive by default, independent of repression by PcG proteins, and may instead be selectively activated by cell-type- or tissue-specific factors [50].

Figure 3 also shows promoter CpG methylation in relation to module membership. DNA methylation in mammalian cells plays multiple roles in cell physiology, including genome stability, repression of endogenous retroviral elements, genomic imprinting. Levels of DNA methylation are dynamically regulated during embroyogenesis but less is known about the role DNA methylation play in gene expression and maintenance of pluripotency in ES cells [51]. Figure 3 shows that methylated genes are significantly under-enriched for black module (*p *= 2.0 × 10^-14^) and significantly under-enriched for blue module genes (*p *= 5.1 × 10^-11^). In Additional File 5, we present the data used for cross-referencing module membership to epigenetic regulators.

#### Variance in *k*_*ME *_Explained by Epigenetic Variables

The above results demonstrate highly significant relationships between module membership and epigenetic variables. In the following, we probe deeper and determine the proportion of variance in module membership that can be explained by the epigenetic variables. Using analysis of variance, we can determine what proportion of the variation in eigengene-based connectivity *k*_*ME *_can be explained by the different epigenetic variables. As can be seen from Table 2, the epigenetic variables explain only 8.3% of the variation in  and 4.2% of the variation in . For , histone trimethylation status (*p *< 2.2 × 10^-16^, prop. of variance explained = 6.7%) and cMyc complex binding (*p *< 2.2 × 10^-16^, prop. of variance explained = 1.5%) explain most of the variation. For , histone trimethylation status (*p *< 2.2 × 10^-16^, prop. of variance explained = 3.4%) and CPG class (*p *= 6.0 × 10^-10^, prop. of variance explained = 0.5%) explain most of the variation. In summary, we find highly significant but relatively weak relationships between module membership and epigenetic variables.

**Table 2:** Module Membership Versus Epigenetic Variables

**Source of Variation in kME**		**kMEblack, Total Prop Var Explained = 8.3%**			**kMEblue, Total Prop Var Explained = 4.2%**		
Source	Degrees Of Freedom	Sums of Sq	Prop. Of Total Var	p-value (F test)	Sums of Sq	Prop. Of Total Var	p-value (F-test)
**Histone Trimethylation (K4, K27, K4&K27, none)**	3	170.91	0.067	< 2.2E-16	30.49	0.034	< 2.2E-16
**cMyc Complex**	1	39.21	0.015	< 2.2E-16	1.48	0.002	2.6E-04
**Oct4 Complex**	1	8.62	0.003	8.0E-08	0.79	0.001	7.5E-03
**CPG class (HCP, ICP, LCP)**	2	4.56	0.002	4.9E-04	4.71	0.005	6.0E-10
**PcG Bound**	1	0.88	0.000	8.7E-02	0.19	0.000	1.9E-01
**CpG Methylated**	1	0.04	0.000	7.1E-01	0.58	0.001	2.2E-02
**Residual Error**	7846	2353.8	0.917		868.36	0.958	

**Total Variation**	7855	2567.11			906.6		

This analysis of variance table reports which epigenetic variables and TF binding data have a significant effect on  (columns on the left hand side) and  (columns on the right hand side). For each variable (source of variation), the columns report the degrees of freedom, the sums of squares, the proportion of total variance explained by the variable and the corresponding F-test p-value. Note that histone trimethylation status is the most significant source of variation for both  and .

### Signed WGCNA Identifies a Pluripotency Module in data from Zhou et al (2007)

To further investigate WGCNA's ability to discover functionally important groups of genes, we turned to an independent data set from Zhou *et al *(2007) [8]. In this study, ES cells were removed from feeder cells and leukemia inhibitory factor (LIF) to induce differentiation. During the course of differentiation, cells were separated based on expression of an Oct4 green fluorescent protein (GFP) reporter gene. Multiple samples were taken from undifferentiated ES cells and cells sorted at days 2, 4, 8, and 15 for high and low Oct4 expression. As before, we first identified gene modules via signed and unsigned methods and then related module membership to external data. In the following we show that a pluripotency/self-renewal and a differentiation module can be found in this new data set. For consistency between data sets, we have colored these modules black and blue, respectively.

#### Cluster Tree Comparison of Unsigned and Signed Networks

**Figure 4:**
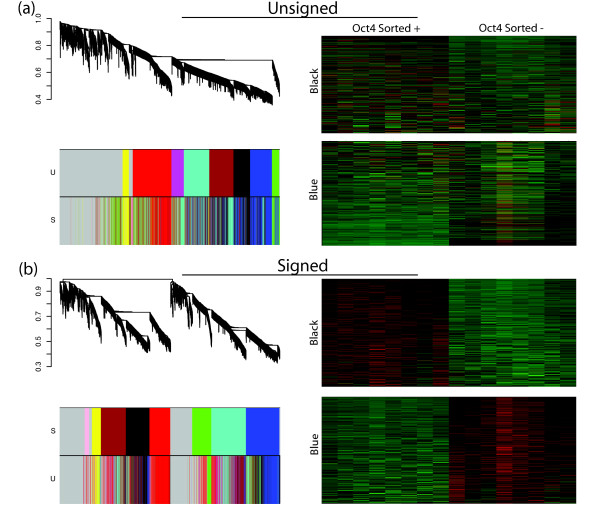
**Unsigned and Signed Networks of the Zhou et al ES Expression Data**. **a, left**, Dendrogram of the unsigned network of the Zhou *et al *(2007) data with color bands below indicating module membership for the unsigned network (U) and the signed network (S). **a, right**, A heat map shows microarray expression profiles accross samples for genes in the blue and black modules in the unsigned network. **b, left**, Dendrogram of the signed network of the Zhou *et al *(2007) data with color bands below indicating module membership for the signed network (S) and the unsigned network (U). **b, right**, heatmap of expression profiles (rows) across samples (columns) for genes in the blue and black modules in the signed network.

Similar to Figure 2, Figure 4 shows the dendrograms of the unsigned and signed networks with color bands indicating module membership in the unsigned and signed networks. The heat maps of the expression profiles of genes in the blue and black modules appear the same across the unsigned and signed networks. The dendrograms, however, reveal network differences. Because module genes roughly stay together, we note that the red, black, blue, and yellow modules are partially preserved in the signed network. However, the black and blue modules are co-located on the same large branch in the unsigned network (Figure 4a) but are separated onto two distinct branches in the signed network (Figure 4b), suggesting a change in network topology. Therefore, depending on branch cutting methods used, the blue and black modules could easily be merged into a larger module in the unsigned network. Indeed, the structure of the dendrogram in Figure 4a suggests that the black and blue modules (along with the turquoise, magenta, and brown) may comprise one large module. For illustrative purposes, we have separated them into smaller modules. In the signed network, on the other hand, the black and blue modules will remain separate regardless of the branch cutting technique employed.

#### Module Membership in Unsigned and Signed Networks

**Figure 5:**
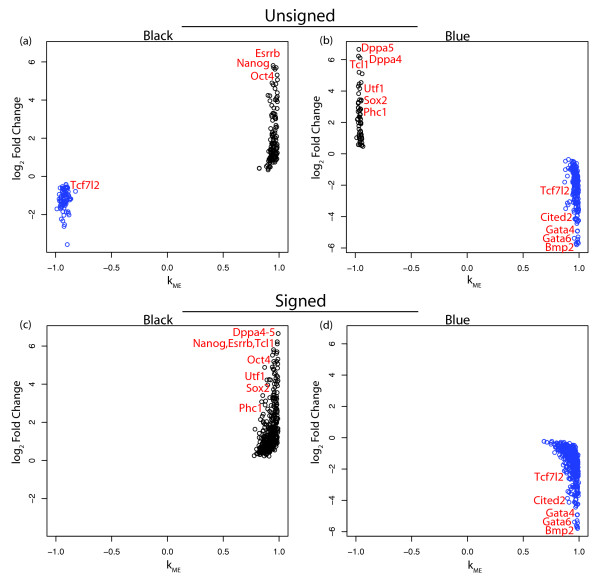
**Expression Changes Versus Module Membership in the Black and Blue Modules (Zhou et al)**. Module membership, *k*_*ME*_, is plotted against *log*_2 _expression fold change (FC) for the black (**a**) and blue (**b**) modules in the *unsigned *network of the Zhou *et al *(2007) data. FC is the ratio between the average expression in Oct4 positive samples and Oct4 negative microarray samples. Known ES cell regulators are labeled. Genes are colored by module membership in the *signed *network. **c **and **d **are analogous to **a **and **b **but module membership is with regard to the *signed *black and blue modules.

Figure 5 shows the module eigengene based connectivity, *k*_*ME*_, of genes in the black and blue modules plotted against their fold change between samples with high and low Oct4 expression as defined in Zhou *et al *(2007). Figures 5a and 5b show that the *unsigned *black and blue modules each contain genes from both the *signed *black and blue modules. However, these genes are clearly separated by fold change and connectivity. Furthermore, both these unsigned modules contain two types of genes, pluripotency/self-renewal related genes and differentiation related genes. For example, the unsigned black module has pluripotency TFs Oct4, Nanog, and Esrrb, while also having Tcf7l2, which is known to play a role in differentiation. The unsigned blue module has pluripotency genes Sox2, Dppa4, Dppa5, Utf1, and Phc1 along with differentiation genes Gata4, Gata6, Cited2, and a second probe for Tcf7l2. Figures 5c and 5d show the black and blue modules in the signed network. These modules contain genes that not only have consistent fold changes and connectivities but also have consistent functional roles. Here the black module contains genes expressed similarly to Oct4 including (Nanog, Sox2, Dppa4, Dppa5, Utf1, and Phc1). Many of the genes found to induce differentiation when knocked down by Ivanova *et al *are in this module (Oct4, Nanog, Sox2, Esrrb, Dppa4, and Tcl1, Fisher's exact test, p-value = 1.01 × 10^-5^). The blue module consists of genes that become expressed when Oct4 is down-regulated upon differentiation and contains many highly connected genes related to ES cell differentiation (Gata4, Gata6, Cited2, Bmp2, Tcf7l2, and Foxa12). These results confirm our earlier finding that the signed black module is a pluripotency module while the signed blue is a differentiation module.

#### A Comparison of Transcription Factor Binding Enrichment in Unsigned and Signed Networks

This conclusion is further supported by our analysis of TF binding patterns in the modules of the Zhou *et al *network. Table 3 shows enrichment for binding by the Oct4 and cMyc groups of TFs and Suz12 from ChIP-seq data from Chen *et al *[16]. In the signed network the black, brown, and red modules are enriched for binding by transcription factors in the Oct4 and cMyc groups and are under-enriched for Suz12 binding. This is not surprising since the black, red, and brown modules are co-located on the same large branch in the signed network (Figure 4b), which shows that they have similar expression profiles and share many of the same gene connections. The blue module, on the other hand, is under-enriched for genes bound by the Oct4 group TFs. These results were also found when using Oct4, Nanog, and PcG binding data from two other studies (Loh *et al *(2006) and Boyer *et al *(2006)) (Additional File 6). The identification of the brown module and the increased enrichment of the black module in the signed network show that signed WGCNA is better at grouping genes into modules based on transcriptional regulation. Furthermore, the black and blue modules are well separated in the signed network (Figure 4b), while in the unsigned network branch cutting could erroneously group them into one large module (Figure 4a).

**Table 3:** Transcription Factor Binding in Zhou et al Networks

**Unsigned Network**				
**Module**	**No. of genes**	**Oct4Complex**	**cMycComplex**	**Suz12**
black	2659	1.55, 2.08e-09	1.4, 4.18e-46	0.666, 6.21e-10
blue	1484	0.872, 0.172	1.09, 0.0125	1.01, 0.447
brown	992	1.2, 0.093	1.02, 0.315	1.11, 0.127
green	1493	0.758, 0.028	0.863, 0.000186	1.36, 4.79e-06
grey	7318	0.766, 1.87e-08	0.803, 1.69e-51	1.2, 7.19e-14
magenta	1583	0.919, 0.28	1.04, 0.147	1.1, 0.0982
red	2593	1.48, 2.3e-07	1.64, 4.02e-107	0.437, 2.49e-26
turquoise	838	0.997, 0.459	1.3, 1.74e-08	0.829, 0.0631
yellow	2002	1.08, 0.213	1.1, 0.00171	0.844, 0.0106

**Signed Network**				
black	1859	1.94, 2.94e-15	1.4, 4.22e-30	0.523, 2.19e-13
blue	1972	0.656, 0.000465	1.03, 0.18	1.13, 0.03
brown	1267	1.45, 0.000755	1.72, 1.22e-59	0.411, 5.78e-14
green	1548	0.818, 0.0763	0.933, 0.0414	1.27, 0.000329
grey	7175	0.729, 1.64e-10	0.784, 2.34e-59	1.24, 2.7e-18
pink	659	0.941, 0.432	1.27, 5e-06	0.474, 2.48e-06
red	2184	1.48, 2.18e-06	1.63, 1.7e-85	0.493, 9.37e-18
turquoise	2317	0.896, 0.156	1.05, 0.0498	1.09, 0.0716
yellow	1870	1.17, 0.0587	1.05, 0.0545	0.885, 0.054

Module enrichments for binding by the Oct4 and cMyc TF Groups amd Suz12. Each enrichment score (before the comma) is followed by its corresponding p-value calculated from the hyper-geometric distribution (uncorrected for multiple comparisons).

### Functional Enrichment Analysis of the Pluripotency and Differentiation Modules

Given that there was significant overlap between the pluripotency modules and differentiation modules of the Ivanova and Zhou networks, we focused further analysis on the network constructed from the Zhou *et al *data set as this network was based solely on differentiation induced expression changes. We determined functional enrichment using the Database for Annotation Visualization and Integrated Discovery (DAVID) [53]. Table 4 shows the significantly enriched GO terms for genes with the 5% highest . In agreement with the finding that the blue module is related to differentiation, genes within this module are significantly enriched for a functional group containing organ development, system development, and cell differentiation (p-value = 0.002). Other highly enriched groups are involved in regulating protein localization (p-value = 9.7 × 10^-17^) and membrane composition (p-value = 5.6 × 10^-8^).

**Table 4:** Functional Pathways in Highly Connected Pluripotency and Differentiation Related Genes in the Zhou et al Network

**p-value**	**Functional Groups**	**Blue Module highly connected genes (kME)**
9.72E-17	er-golgi transport; protein localization; protein transport; vesicle-mediated transport; secretion by cell; cellular localization; secretory pathway; intracellular transport;	Myl6 (0.994), Sh3glb1 (0.993), Tm9sf3 (0.993), Tram1 (0.992), Derl1 (0.991), Serinc1 (0.991), Lman1 (0.991), Lrp10 (0.991), Mcfd2 (0.99), Mcfd2 (0.99), Tmed10 (0.99), Tpcn1 (0.989), Arl1 (0.989), Tinagl (0.987), Rab2 (0.987), Txndc1 (0.987), Col4a1 (0.987)
5.65E-08	Glycan structures – biosynthesis 1; signal-anchor; transferase activity, glycosyltransferase	Glt8d1 (0.993), Creb3 (0.991), Fut8 (0.99), Fkrp (0.99), Extl2 (0.989), Glt8d3 (0.987), Itm2c (0.986), Hs3st1 (0.986), Pofut2 (0.986), Dpagt1 (0.985), Mgat2 (0.983), Abhd6 (0.982), Ddost (0.982), Ndst2 (0.981), B4galnt1 (0.981), St3gal6 (0.98)
2.98E-06	membrane; transmembrane; transmembrane region; topological domain:Cytoplasmic	H13 (0.996), Pdgfra (0.994), Cd59a (0.994), Glt8d1 (0.993), Sh3glb1 (0.993), Tm9sf3 (0.993), Tram1 (0.992), Gdpd5 (0.991)
0.00185	organ development; system development; anatomical structure morphogenesis; cell differentiation; organ morphogenesis	Pdgfra (0.994), Myl6 (0.994), Sh3glb1 (0.993), Lmo4 (0.992), Rgnef (0.989), Syvn1 (0.988), Kit (0.988), Fndc3b (0.988), Txndc1 (0.987), Lama1 (0.987), Barx1 (0.986), Col4a2 (0.986), Ctgf (0.985), Fgf3 (0.985), Crim1 (0.983), Pthr1 (0.983)

**p-value**	**Functional Groups**	**Black Module highly connected genes (kME)**

1.98E-08	response to DNA damage stimulus; DNA damage; DNA repair	Msh6 (0.993), Rif1 (0.983), Mre11a (0.982), Setx (0.974), Xrcc5 (0.971), Chek1 (0.968), Xab2 (0.967), Xrn2 (0.967), Trp53 (0.959), Npm1 (0.958), Tdp1 (0.955), Bccip (0.954)
3.75E-08	Mitochondrion; transit peptide; Mitochondrion	Mrpl15 (0.992), Ppif (0.991), Mrps5 (0.987), Hspa9 (0.984), Coq3 (0.984), Tst (0.981), Mrpl45 (0.98), Akap1 (0.979), L2hgdh (0.978), Mrps31 (0.978), Chchd4 (0.976), Abce1 (0.975), Dci (0.975), Fpgs (0.974), Mrpl39 (0.973), Bdh1 (0.971)
5.83E-08	nucleus; biopolymer metabolic process; DNA binding; cellular metabolic process; Transcription regulation;	Msh6 (0.993), Pes1 (0.991), Zic3 (0.991), Uchl1 (0.99), Rnf138 (0.99), Rnf138 (0.99), Wdr36 (0.989), Pou5f1 (0.989), Rbpj (0.987), Glo1 (0.987), Tdgf1 (0.987), OTTMUSG00000010173 (0.986), Aarsd1 (0.986), Nup133 (0.985), Xpo1 (0.985), Xpo1 (0.985), Dnajc6 (0.985), Klhl13 (0.984), Dppa4 (0.984),
5.26E-04	cell cycle phase; cell cycle process; cell cycle; mitotic cell cycle; mitosis; cell division	Pes1 (0.991), Rif1 (0.983), Mre11a (0.982), Gtpbp4 (0.972), Chek1 (0.968), Mnat1 (0.966), Rcc2 (0.964), Gadd45gip1 (0.963), Rpa1 (0.961), Hells (0.96), Trp53 (0.959), Terf1 (0.959)

Enriched GO terms of genes with the highest 5% black and blue *k*_*ME*_'s. Highly connected genes in each functional group are given. All *k*_*ME *_values are highly significant (Bonferroni corrected p-value < 0.00015).

Table 4 also shows significant GO terms for genes with the 5% highest . Given that many pluripotency TFs are in the black module, it is not surprising that the functional classifications, DNA binding and transcriptional regulation, are significantly enriched (p-value = 5.4 × 10^-8^). However, two functional classifications, DNA damage/repair and mitochondrial function, are more significantly enriched than the transcriptional regulation group (p-values = 2.0 × 10^-8 ^and 3.8 × 10^-8^, respectively) suggesting that these pathways play important roles in maintaining pluripotency and self-renewal.

We also used Ingenuity Pathway Analysis, IPA, to compare functional enrichment in the pluripotency and differentiation modules (Ingenuity Systems, ). Additional File 7 shows that functional groups similar to those found using DAVID are enriched in the black and blue module respectively. Cell cycle and DNA replication, recombination, and repair are enriched in the black module compared to the blue module and skeletal, muscular, and cardiovascular system development are enriched in the blue module.

### Comparison to a Standard Differential Expression Analysis

Here we compare some of our WGCNA results with those of a standard differential expression analysis. In Figure 2c and Figure 5 we showed that for some modules a strong relationship between module membership (*k*_*ME*_) and differential expression (gene significance/fold change) can be observed. In [33], we provide a geometric description of modules for which such a relationship can be observed. While a close relationship may exist between *k*_*ME *_and a Student t-test statistic, it does not imply that corresponding gene ranking procedures are equivalent.

Here we compare signed WGCNA to standard differential expression methods using three different approaches. First, we show that a gene ranking based on *k*_*ME *_is more consistent (reproducible) than that based on the Student t-test in our data. Specifically, we computed two gene rankings for the Ivanova *et al *data set, one ranked by t-statistic and the other by connectivity to a module of interest. We similarly computed two such rankings for the Zhou *et al *data set and studied the overlap between the two data sets (Additional File 8). Of the 1000 genes most significantly down regulated upon differentiation in each data set 139 overlap (hyper-geometric p-value = 1.0 × 10^-20^). However, when ranking genes by connectivity to each data set's pluripotency model there is an increase in overlap to 230 (p-value = 1.7 × 10^-75^, Additional File 8). This increased consistency is also seen in genes up regulated upon differentiation where 77 genes overlap between the two data sets (p-value = 0.02) when ranking by t-statistic and 161 genes overlap when ranking by connectivity to the differentiation modules (p-value = 2.8 × 10^-31^, Additional File 8).

**Figure 6:**
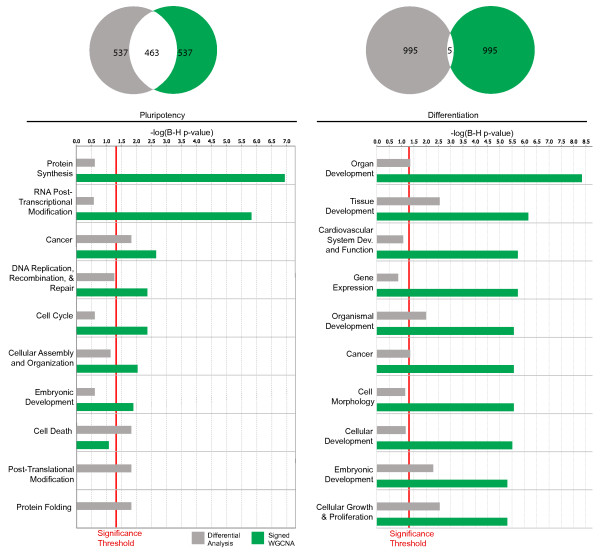
**Comparison of Genes Ranked by Network Connectivity and Differential Expression in the Ivanova et al data set**. Ingenuity Pathway Analysis of functional enrichments in the set of genes ranked within the top 1000 by Student's t-test and *k*_*ME *_and yet do not overlap with each other. Venn diagrams show the amount of gene overlap between the top 1000 black (pluripotency) module genes and the top 1000 genes most significantly down-regulated upon Oct4 RNAi (left); gene overlap between the top 1000 blue (differentiation) module genes and the 1000 genes most significantly up-regulated with Oct4 RNAi (right). Significance of differential expression was determined using Student's t-statistic. p-values have been corrected for multiple hypothesis tests (Benjamini-Hochberg). Only significantly enriched functional groups are shown.

A second approach for comparing gene rankings is to use the functional enrichment with regard to known gene ontologies. To compare the abilities of *k*_*ME *_versus the conventional t-statistic in identifying functionally interesting groups of genes we consider functional enrichment of genes found by one ranking method but not the other (Figure 6). Of the 1000 genes most strongly connected to the Ivanova *et al *pluripotency module (black), 463 overlap with the 1000 genes most significantly down regulated upon Oct4 RNAi in the same data set. Figure 6 shows the functional enrichment from Ingenuity Pathway Analysis, IPA, of the 537 genes in each group that do not overlap. Genes found only by signed WGCNA are significantly enriched for functions important to ES cells like DNA Replication, Recombination, and Repair, Cell Cycle, Cancer, Protein Synthesis, and RNA Post-Transcriptional Modification, which have previously been found using network methods [54]. Generally, genes identified by signed WGCNA exhibit more significant enrichment of functional classifications than those found by standard differential analysis.

We similarly compare the 1000 most highly connected genes in the differentiation (blue) module and the 1000 genes that are most significantly up regulated upon Oct4 RNAi in the Ivanova network. Interestingly, only five genes overlap (Figure 6). By examining those genes that do not overlap we see that ranking by connectivity yields greater significant enrichment for many functional groups important in ES cell differentiation including Organ Development, Tissue Development, Cell Morphology etc.

Similar analysis of pluripotency genes in Zhou *et al *yields consistent results with DNA Replication, Recombination, and Repair being more enriched when ranked by connectivity (Additional File 9) while analysis of highly connected genes in the differentiation module shows that differential analysis moderately out performs ranking by connectivity. The differences in functional enrichment in the Zhou *et al *data set are subtle given that there is more overlap between the two rankings (Additional File 8). This large overlap is likely due to the simplicity of the expression array samples which are filtered into only two groups, those that exhibit Oct4 expression and those that do not. Meanwhile, signed WGCNA is especially useful in Ivanova *et al *where smaller overlap is caused by the complexity of the expression samples which are made of many different RNAi treatments.

A third approach for comparing gene rankings is to use the enrichment with regard to epigenetic and transcriptional regulators. In Additional File 4 we relate different gene rankings to enrichment significance with regard to the following variables (a) histone H3K4 alone versus all others, (b) bivalent H3K4 & H3K27 versus all others [50], (c) High CPG class versus all others (i.e. HCG versus ICG and LCG), (d) promoter CPG methylation status [51], (e) Oct 4 complex binding status, (f) cMyc complex binding status. We report results for 3 different gene rankings using the Ivanova data: the black and blue curve represent gene rankings according to  and , respectively. The grey curve represents ranking according to a Student T-test of differential expression. Additional File 4 shows that black and blue module genes can have very different enrichment results that tend to be very different from those of a standard analysis. This analysis illustrates how module membership provides important complementary variables along the Student t-test for understanding differences between genes.

The increased functional enrichment and improved consistency between data sets suggest that signed WGCNA is a complementary method to standard differential analysis. In practice, we recommend to use both *k*_*ME *_and the Student t-test to find highly differentially expressed intramodular hub genes.

### Pluripotency Module Genes involved in Transcriptional Regulation and Chromatin Structure

**Figure 7:**
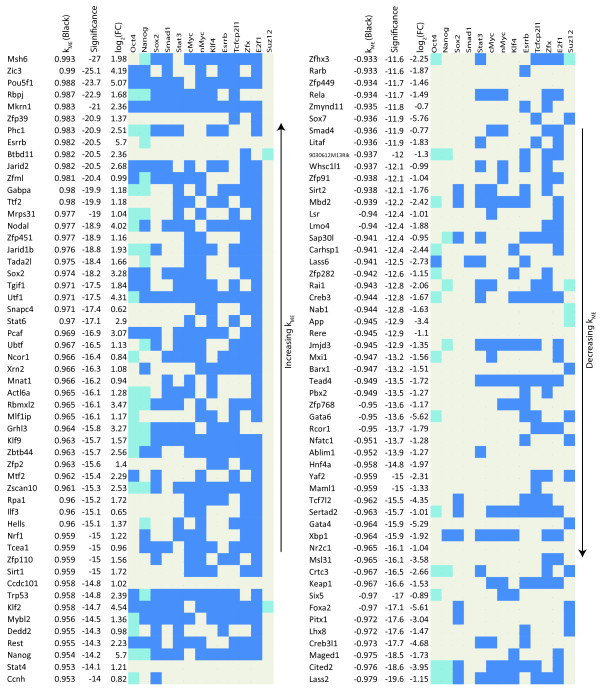
**Transcriptional Regulators related to Pluripotency and Differentiation in the Zhou Network**. TF and Suz12 binding in the promoter regions of highly connected genes related to ES cell pluripotency and self-renewal with GO terms of transcriptional regulation or chromatin structure. Genes are listed by black *k*_*ME *_(positive, left and negative, right) along with their corresponding significance level (*log*_10 _of the Bonferroni corrected p-value generated by a correlation test). Binding data from Chen *et al *(2008), Boyer *et al *(2006), and Loh *et al *(2006), are marked in blue (bound) and beige (unbound). For Oct4, Sox2, and Suz12, where binding is given by two studies, binding will be blue if it is found in both studies and light blue if found in only one.

To gain a better understanding of the regulatory network involved in maintaining ES cell pluripotency and self-renewal, we first looked at genes with high black  and GO terms related to transcriptional regulation or chromatin structure. Figure 7 lists such genes and contains information about how these genes are bound by TFs in the Oct4 and cMyc groups, and Suz12 plus Klf4, Esrrb, and Tcfcp2l1. The sign of black  allows us to distinguish genes that promote pluripotency from those that repress it. As expected, many of the genes with high positive module membership measure () are known to participate in ES cell regulation (Zic3, Mkrn1, Phc1, Esrrb, Jarid2, Nodal, Jarid1b, Tgif1, Utf1, Hells, and Rest) [20,55,56]. Importantly, four TFs capable of reprogramming differentiated cells into a ES cell like state are in this list (Sox2, Klf2, Nanog, and Pou5f1(Oct4)) [10,11], confirming that the black module captures the known core transcriptional regulatory network responsible for maintaining a cell's stemness. Genes with high positive values of black module membership () but have not been implicated in maintaining pluripotency and self-renewal should therefore be strong candidates for further functional study. Such genes include Msh6 (involved in DNA damage and repair [57]), Rbpj (involved in Notch signaling [58]), Zfp39 (spermatogenesis [59]), and Nrf1 (mitochondrial organization and biogenesis [60]). Similarly, many of the highly negatively connected genes in the black module are known to play a role in ES cell differentiation (Cited2, Gata4, Gata6, Tead4, Foxa2, and Sox7). Because  and  are highly negatively correlated (*r *= -0.99), genes with high positive  have negative  and vice versa. Highly negatively connected genes that are not known to be involved in ES cell differentiation are also candidates for functional investigation. Some genes like Maged1 are known to play a role in cell differentiation but have not been shown to be important in ES cell differentiation specifically [61]. Other genes like Lass2 have little known about their role in cell differentiation. Figure 7 also shows that genes positively connected to the black module tend to be bound by more ES cell related TFs compared to the negatively connected genes, supporting the idea that these TFs bind and activate pluripotency and self-renewal genes [13].

### Pluripotency Module Genes not Involved in Transcriptional Regulation or Chromatin Structure

**Figure 8:**
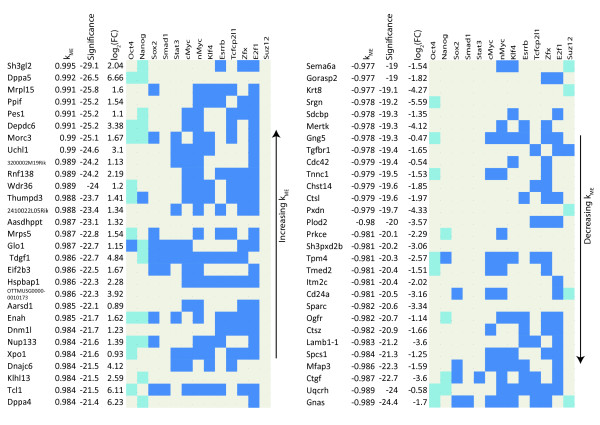
**Non-Transcriptional Regulators Related to Pluripotency and Differentiation**. TFs and Suz12 binding of highly connected genes related to ES cell pluripotency and self-renewal lacking GO terms for transcriptional regulation or chromatin structure. Genes are tablulated in the same format as Figure 7.

Genes that are not involved in transcriptional regulation or chromatin modification (as defined by GO analysis) but have high average  are also of interest. Figure 8 lists such genes along with TF and Suz12 binding information, connectivity, and fold change. Once again the importance of some genes has been validated, while others should be candidates for further research. Genes like Dppa5, Dppa4, and Tcl1 are markers of pluripotency and Nup133, a nuclear pore complex subunit, has recently been shown to be necessary in the maintenance of pluripotency [62]. Nup133 highlights the usefulness of signed WGCNA. Using the t-statistic from standard differential analysis Nup133 is ranked 222^*th *^most significantly down regulated upon differentiation while using connectivity its rank moves to 28^*th*^. Other candidate genes include Sh3gl2, which binds lipids and proteins [63], Mrpl15, a mitochondrial ribosomal protein, and Ppif, involved in mitochondrial function and oxidative stress-induced cell death [64]. Of the negatively connected genes in Figure 8, Ctsl (cathepsin L) has recently been shown to cleave the histone H3 N-terminus during ES cell differentiation [65], while little is known about Ctsz, also a cathepsin, while Gnas and Ctgf are differentiation genes [66,67]. The high negative connectivity of Uqcrh, a mitochondrial inner membrane protein [68], along with the high positive connectivity of Mrpl15 and Ppif, confirms that mitochondrial regulation may be distinct in ES cells [69-71] and suggests that they may be important regulators of mitochondrial function in ES cells.

### Pluripotency Module Genes that Lack Binding by Known Pluripotency TFs

**Figure 9:**
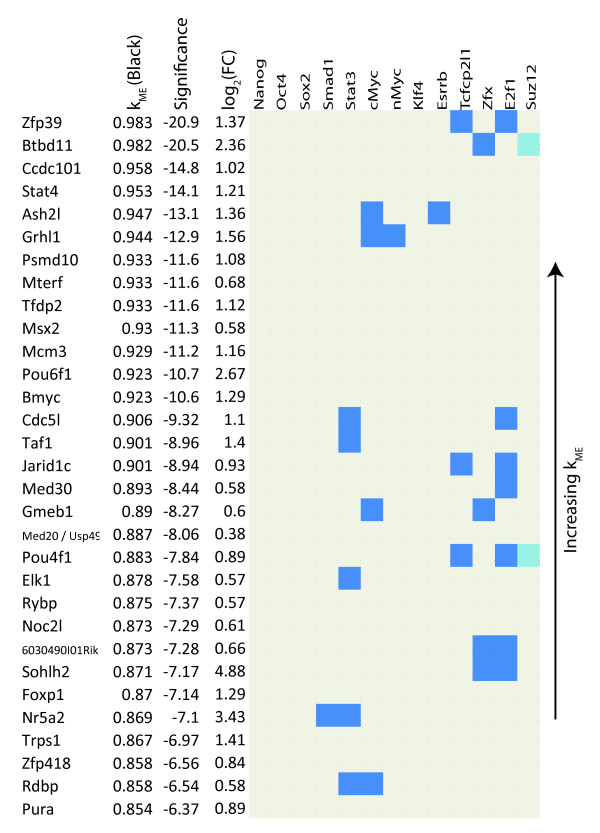
**Pluripotency Transcriptional Regulators that are not Bound by the Core TF Machinery in ES Cells**. Genes related to ES cell pluripotency and self-renewal with GO terms of transcriptional regulation or chromatin structure and little pluripotency TF binding. Genes are listed by black *k*_*ME *_along with their corresponding significance level (*log*_10 _of the Bonferroni corrected p-value generated by a correlation test). Binding data from Chen *et al*, Boyer et al and Loh *et al*, are marked in blue (bound) and beige (unbound). For Suz12, light blue indicates that a genes is called bound in Chen *et al *or Boyer *et al *but not in both.

Figure 9 shows genes with relatively high  that lack binding by the TFs Nanog, Oct4, Sox2, and Klf4 [10,11] and have low binding (≤ 2) by other TFs that maintain pluripotency (Smad1, Stat3, cMyc, nMyc, Esrrb, Tcfcp2l1, Zfx, and E2f1). Transcriptional regulators that have high positive module membership in the black module but lack binding by the pluripotency TFs are of interest since their strong module membership cannot be explained via regulation by these TFs. Their high average  suggests that they may be upstream regulators of Oct4, Sox2, Nanog, and other genes important to pluripotency.

To further investigate the role of these genes, we used motif scanning methods described in Zhou *et al *(2007) [8] to determine if the binding sites of these genes are contained in regions co-bound by TFs in the Oct4 group or cMyc group in ChIP-seq data from Chen *et al *(2008). We concentrated solely on Lrh1 (Nr5a2) and Elk1 since their motifs have known position specific weight matrices while the other genes lack known motifs. Table 5 shows the enrichment and significance of motifs scanned. Sox2 and Oct4 bind to the composite SoxOct motif besides their own, Stat3 binds the Stat1 motif, and cMyc binds the Ebox motif. Both the Oct4 and cMyc groups' expected motifs are enriched. For example, the SoxOct motif has over three fold enrichment in regions bound by TFs in the Oct4 group. Interestingly, the Lrh1 motif is more enriched than the Nanog motif in sequences bound by the Oct4 group, which contains Nanog binding by definition. This reinforces the hypothesis that Lrh1 co-binds regions bound by TFs in the Oct4 group [8]. Furthermore, Lrh1 sites are found in the promoter regions of Pou5f1 (Oct4), Klf4, Dppa5, and Suz12 with Pou5f1 having three separate sites. These motif sites and Lrh1's known importance in ES cells, suggest that it may be an upstream regulator of these pluripotency factors and as such is a candidate for experimental validation [72]. The Elk1 motif is also significantly enriched in sequences bound by the cMyc group, thus Ekl1 may co-regulate genes bound by TFs in this group.

**Table 5:** Motif Enrichment in Genes bound by Oct4 or cMyc TF Groups

		**Oct4 Group**			**cMyc Group**		
**Motif**	**Binding TF**	**No. of genes**	**ratio**	**p-value**	**No. of genes**	**ratio**	**p-value**
SoxOct	(Oct4, Sox2)	66	3.41	2.0E-21	103	0.45	6.4E-24
Oct4	(Oct4)	37	2.33	9.9E-08	151	0.81	2.5E-03
Sox2	(Sox2)	51	3.06	3.8E-15	178	0.86	1.4E-02
Nanog	(Nanog)	18	2.20	5.0E-04	122	1.35	3.9E-04
Stat1	(Stat3)	34	2.83	7.3E-09	145	1.32	1.0E-04
Ebox	(cMyc)	6	0.59	9.5E-01	373	4.02	2.6E-207
E2f1	(E2f1)	1	1.44	9.4E-02	284	2.25	1.2E-40
Klf4	(Klf4)	38	1.02	4.0E-01	235	0.61	1.4E-19
Lrh1	(Lrh1)	28	2.22	1.4E-05	158	1.12	6.9E-02
Elk1	(Elk1)	10	0.19	3.1E-02	170	1.64	8.6E-11

Sequences co-bound by all TFs of the Oct4 group (Oct4, Sox2, Nanog, Smad1, and Stat3) or all TFs of the cMyc group (cMyc, nMyc, E2f1, and Zfx) in the Chen *et al *data set were scanned for relevant motifs, plus the Lrh1 (Nr5a2) and Elk1 motifs. There were 122 genes bound by all TFs in the Oct4 group and 1173 genes bound by all TFs in the cMyc group. Enrichments, computed by comparing against motif scans in control sequences, and p-values are shown below.

### A Geometric Interpretation of Signed WGCNA Modules

To understand how signed WGCNA is better able to separate genes into functional modules in the Ivanova data set, we plotted genes in the signed black or turquoise module relative to the unsigned turquoise module eigengene (Additional File 10). Note that genes located in the black and turquoise modules in the signed network are clearly separated into two clusters. Because a module eigengene is defined as the first principle component of its module, it describes the main direction in which the module's gene expressions vary. Note that the signed module eigengenes are oriented in the direction of their clusters. The direction of the unsigned turquoise module eigengene is more difficult to interpret. Because the turquoise module in the unsigned network contains two distinct signed modules (black and turquoise), its module eigengene describes the variance between these two sub-modules and the variance within the larger sub-module, the signed turquoise. As such, the unsigned turquoise module eigengene fails to quantify the true importance of highly connected genes in the signed black module. For example, Oct4's  in the unsigned turquoise module is -0.74 while it is 0.94 in the signed black module. Thus, Oct4 is not identified as a hub gene in the unsigned network while it is clearly a hub gene in the signed network.

## Conclusion

We show that a systems biology approach, which utilizes gene expression, transcription factor binding, genomic, epigenetic and gene ontology data, can be improved by accounting for the sign of co-expression relationships. We also show that signed WGCNA has advantages over standard differential expression methods. Specifically, signed WGCNA has more consistent gene rankings between data sets (see Additional File 8), is better able to identify functionally enriched groups of genes (Figure 6), and its focus on module eigengenes circumvents the multiple testing problems that plague standard gene-based expression analysis. Below, we highlight several novel stem cell related genes that would not have been found using a standard differential expression analysis.

Signed WGCNA provides novel insight into murine ES cell biology, which unsigned WGCNA is unable to provide. Applying these signed methods to previously published data, we identified pluripotency and differentiation gene modules not found in unsigned networks or differential analysis. The results of signed WGCNA are robust as it identifies similar modules in independently published data sets. We show that module eigengene based connectivity *k*_*ME *_is valuable for annotating genes with regard to module membership and for identifying genes related to pluripotency and differentiation. As a resource, we provide a module membership annotation for each gene with regard to the signed modules (Additional Files 11 and 12).

Many current studies focus on the role transcriptional regulators play in ES cell maintenance. As expected, the pluripotency module is enriched with genes active in transcriptional regulation, e.g. Oct4, Sox2, Klf2, Nanog, Jarid1b, Jarid2, Nodal, Tgif1, and Esrrb, and contains other genes expected to play a role in ES cell function, such as Dppa4 and Dppa5. The module also contains genes that have recently been shown to be necessary for maintaining the pluripotent state, Nup133 and Utf1 [45,62].

Interestingly, the pluripotency module contains genes with roles in two other pathways, DNA repair and mitochondrial function, which are not found by standard differential analysis. The enrichment for genes that respond to DNA damage is not surprising given that ES cells spend a larger portion of their cell cycle in S phase and have a shorter G1 phase than differentiated cells [73]. An emphasis on accurate DNA replication is expected since it helps ES cells maintain a stable genome and prevents errors from being inherited by differentiated cells. Mitochondria in ES cells may assist in the prevention of DNA damage [71]. During aerobic production of adenosine triphosphate (ATP), mitochondria leak superoxides leading to the creation of reactive oxygen species (ROS), which damage DNA. ES cells, however, produce ATP anaerobically and thus minimize the amount of DNA damaging ROS [69,71]. ES cells also have fewer mitochondria than differentiated cells and their mitochondria are smaller, have fewer cristae, lack dense matrices, and are perinuclearly located [69-71]. Our use of signed WGCNA reveals that in addition to genes involved in transcriptional regulation, genes that prevent or repair DNA damage are key to maintaining pluriotency and self-renewal.

Figure 3 reports significant relationships between module membership, chromatin structure and epigenetic modifications (histone modifications and DNA methylation), which are known to play a role in controlling gene expression during ES cell self-renewal and differentiation. While the relationships are highly significant, we find that epigenetic variables and binding data explain only 8.3% of the variation in module membership  and 4.3% of the variation of  (Table 2). In Additional File 5, we provide gene annotations with regard to module membership, transcription factor bindings, histone trimethylation status, CpG DNA methylation etc.

Using module eigengene based connectivity  we find that many known differentiation related genes are highly connected in the differentiation (blue) module, Cited2, Gata4, and Gata6, along with Ctsl, which has recently been shown to be active in differentiation [65]. We also find that Uqcrh, a gene involved in the electron transport chain, is highly connected in this module, lending support to the argument that ES cell mitochondria differ from those in differentiated cells. Module eigengene based connectivity enabled us to identify novel candidate genes in the differentiation module, like Uqcrh, that warrant experimental validation (Figure 8). For the pluripotency module interesting candidate genes are Msh6, Ppif, Sh3gl2, Rbpj, Elk1, Nrf1, Nup133, Mrpl15, and Zfp39 (Figures 7 and 8). These genes lack significant fold change but are highly connected and thus would not be found using standard differential analysis. Using sequence data with motif analysis we confirm the importance of two genes, Nr5a2 and Elk1, computationally.

We use gene ontology information and literature results to provide strong statistical evidence that these candidate genes are very promising and justify further biological study. Our article provides a resource in form of module based gene annotation tables that could form the starting point of future biological validation studies. Depending on their function, these candidate genes can be tested by RNAi knock down, viral infection in order to increase the efficiency of reprogramming, or, if they bind DNA, analyzing their binding sites. Our article demonstrates that signed WGCNA not only identifies many well known ES cell regulators; it also yields novel insights regarding ES cell function.

## Methods

Our statistical methods are implemented in the WGCNA R software package [34]. For example, a signed network using the power *β *= 12 is constructed with the R command *ADJ *= *adjacency (datExpr, power = 12, type = "signed")*.

### The Topological Overlap Matrix

The topological overlap, reflects the relative interconnectedness between genes *i *and *j*. It takes into account the relationship between the two genes and their shared connection pattern to other genes [19,35-37]. The topological overlap between two genes is defined as follows:

(6)

where *a*_*ij *_is the above defined adjacency, *l*_*ij *_= ∑_*u*≠*i*,*j *_*a*_*iu*_*a*_*uj*_, and *k*_*i *_= ∑_*u*≠*i *_*a*_*iu*_.

### The Effect of the Co-expression Similarity Measure on the Topological Overlap Measure

The choice of co-expression similarity measure (i.e.  versus ) has strong implications for the resulting topological overlap measure. Note that *cor*(*x*_1_, *x*_*u*_) = -1 implies  and . In case of a soft threshold *β *= 1, we find the following corresponding weighted adjacency measures:  and . In the following, consider the simple network in Additional File 1 (part a) where adjacencies *a*_1*u *_= *a *and *a*_*u*2 _= *a *for *u *= 3, ..., *n*. Using equation (6), *t*_12 _simplifies as follows:



where the latter approximation assumes that the number of genes *n *is large. For the above situation (with *β *= 1) this implies  ≈ *a*^*unsigned *^= 1 and  ≈ *a*^*signed *^= 0. Thus, the two genes have high interconnectedness in an unsigned network but zero interconnectedness in a signed network. Additional File 1 (part b) shows a simple network where genes 1 and 2 are oppositely correlated with their neighbors. Here the choice of gene co-expression measure results in a very different topological overlap measures, which in turn, leads to different modules.

### The Module Eigengene and Module Membership

For the *q*-th module, we summarize its expression data, by a module eigengene, *E*^(*q*)^, found by singular value decomposition of the expression data [39]:

(7)

where *X*^(*q*) ^is the *n*^(*q*) ^× *m *matrix of standardized expression profiles of the *n*^(*q*) ^genes in the module across *m *samples, *U*^(*q*) ^is an *n*^(*q*) ^× *m *matrix with orthogonal columns, *D*^(*q*) ^is an *m *× *m *diagonal matrix of singular values, and *V*^(*q*) ^is an *m *× *m *orthogonal matrix of singular vectors. Then *E*^(*q*) ^is defined by:

(8)

where  is the singular vector in *V *^(*q*) ^corresponding to , the largest absolute singular value in *D*^(*q*)^. The module eigengene, *E*^(*q*)^, can be used to define the module eigengene based connectivity, *k*_*ME*_, or fuzzy module membership [25,27,33,39] via

(9)

### The Relationship between *k*_*i *_and *k*_*ME, i*_, when *β *= 1

To study the relationship between module eigengene based connectivity, *k*_*ME, i*_, and intramodular connectivity, *k*_*i*_, we consider a special case of a signed weighted network with *β *= 1 (i.e. ). Then the intramodular connectivity is given by



where *n*^(*q*) ^is the number of genes in the *q*^*th*^module. It has been shown that network modules are approximately factorizable (i.e. *cor*(*x*_*i*_,*x*_*j*_) ≈ *cor*(*x*_*i*_,*E*^(*q*)^) × *cor *(*x*_*j*_,*E*^(*q*)^)) [33,39]). This approximation implies

(10)

where *E*^(*q*) ^is the module eigengene of module *q*, , and . Equation (11) implies an approximate linear relationship between  and  if *β *= 1. Using real data, we illustrate this relationship in Additional File 2.

### Motif Enrichment

Methods developed in Zhou *et al *[8] were used to scan sequences for motifs with pre-defined position specific weight matrices. Sequences were determined by extending bound ChIP-seq sites 150 bp up and downstream resulting in regions approximately 330 bp long. Any overlapping regions were then joined into larger meta-regions. A set of control sequences was scanned to determine a motif enrichment ratio. The control group was created by randomly sampling 5,000 probes from the Agilent Mouse Promoter Whole Genome ChIP-on-chip Microarray Set. These probes are distributed -5.5 kb upstream to +2.5 downstream of approximately 17,000 known gene transcription start sites from UCSC's version mm8 genome.

Overlapping control probes were merged into meta-regions as described above. Enrichment of a motif is defined as  where *M*_*T *_is the number of observed sites and *λ *is the number of expected sites, , where *M*_*C *_is the number of sites in the control, *N*_*C *_the length of all control sequences, and *N*_*T *_the length of all bound sequences. Statistical significance is determined by the Poisson distribution with *λ *as the mean.

## Authors' contributions

MJM carried out the study, analyzed the data, and drafted the article. KP and GF provided embryonic stem cell expertise in interpreting data. QZ provided guidance in statistical analysis. SH conceived of the study and assisted in its design and data analysis. All authors helped write the article.

## Supplementary Material

Additional file 1 **A Simple Illustration of How the Choice of a Similarity Measure Affects TOM**. The TOM measure of interconnectedness is often used to define clusters of highly interconnected genes. Here we use very simple networks to highlight properties of the TOM measure. (a) Computing the topological overlap between genes 1 and 2 when all connection strengths between intermediate genes equal the constant *a*. (b) The numbers on the edges of the left network are correlations while the numbers on the edges of the networks on the right hand side equal corresponding unsigned adjacencies (upper network) and signed adjacencies (lower network). In a signed network, the topological overlap between genes 1 and 2 is very low because intermediate genes 3 and 4 have negative correlations with gene 1. In contrast, the topological overlap between genes 1 and 2 is high in an unsigned network. Click here for file

Additional file 2 **Intramodular Connectivity is Highly Correlated with Module Eigengene Based Connectivity *k*_*ME*_**. For each module from the Zhou et al data, we plot intramodular connectivity (defined using a weighted network with power *β *= 1) versus module eigengene based connectivity *k*_*ME*_. We find that the two connectivity measures are highly correlated. A theoretical derivation between network concepts and eigengene based analogs is presented in [32]. Click here for file

Additional file 3 **Binding Enrichments for Unsigned and Signed Ivanova et al (2006) Networks**. This file contains enrichments and corresponding p-vaules for binding from Loh *et al *(2006), Boyer *et al *(2007), and Chen *et al *(2008). Click here for file

Additional file 4**Comparing Gene Rankings to Regulators of Gene Expression**. Here we relate different gene rankings to enrichment significance with regard to the following variables (a) histone H3K4 alone versus all others, (b) bivalent H3K4&H3K27 versus all others [49], (c) high CPG class versus all others (i.e. HCG versus ICG and LCG), (d) promoter CPG methylation status [50], (e) Oct 4 complex binding status, (f) cMyc complex binding status. We report results for 3 different gene rankings using the Ivanova data: the black and blue curve represent gene rankings according to  and , respectively. The grey curve represents ranking according to a Student T-test of differential expression. Additional File 4 shows that black and blue module genes can have very different enrichment results that tend to be quite different from those of a standard analysis.Click here for file

Additional file 5 **Data for Cross-Referencing Module Membership to Epigenetic Regulators**. In this Additional File, we merged Additional File 11 (Module Membership *k*_*ME *_etc in the Ivanova data) with a Table (S4) from [50] that contained promoter CPG methylation and lysine trimethylation data from [49]. This Additional file reports module membership values, histone modifications, promoter CpG Status, and polycomb, Oct4 complex, cMyc complex binding etc. The table reports data regarding genes whose promoters have H3K4me3, H3K27me3, both, or neither histone mark. Further, column Class reports CPG promoter classifications (high HCP, intermediate ICP, or low LCP). The column Methylated reports which genes are known to be methylated (value 1) or unmethylated (0) [50]. For completeness, it also includes information regarding genes bound by Nanog or Oct4 in the proximal promoter (within 10 kb) or bound by Nanog and Oct4 long range (within 500 kb), or bound by Polycomb [5,6]. Click here for file

Additional file 6 **Binding Enrichments for Unsigned and Signed Zhou et al (2007) Networks**. This file contains enrichments and corresponding p-values for binding from Loh *et al *(2006), Boyer *et al *(2007), and Chen *et al *(2008). Click here for file

Additional file 7 **Ingenuity Pathway Analysis of the Pluripotency and Differentiation Modules from Zhou et al (2007).** Click here for file

Additional file 8 **Comparison of Overlap in Ivanova et al and Zhou et al (2007) when Ranking by t-statistic and Connectivity.** Click here for file

Additional file 9 **Ingenuity Pathway Analysis of Genes Ranked by Connectivity and Differential Expression in Zhou et al (2007).** Click here for file

Additional file 10 **Understanding Signed Module Membership**. Here we visualize the relative position of unsigned and signed similarity modules. We used module eigengene based connectivity, *k*_*ME *_= *cor*(*x*_*i*_, *E*), to visualize a gene's module membership, focusing on genes located in the *signed *turquoise or black modules. For any vectors *a *and *b *with angle *θ *between them, their correlation can be interpreted as *cor*(*a*, *b*) = *cos*(*θ*). Using this relationship we plotted genes in polar coordinates (radially) relative to the *unsigned *turquoise module. The figure shows the angle, *θ*, between the gene's expression profile and the turquoise module eigengene from the *unsigned *network, indicated by the solid turquoise line. Each gene's radius is defined as the absolute value of its *log*_2 _expression fold change (FC). FC is the ratio between the average expression in the control RNAi samples and the average expression in the Oct4 RNAi knock down samples. For reference the *signed *turquoise and black module eigengenes are indicated by dashed turquoise and black lines, respectively, and genes are colored by *signed *module membership. Known ES cell regulators and differentiation markers are labeled. Note that the signed module eigengenes (dashed lines) reflect the relationship *within *their corresponding modules while the unsigned module eigengene (solid line) reflects the relationship *between *the two signed modules. Click here for file

Additional file 11 **Module Membership and Binding Information in the Signed Ivanova et al (2006) Network**. This file contains module membership, *k*_*ME*_, and binding data from Loh *et al *(2006), Boyer *et al *(2007), and Chen *et al *(2008) for each gene on the microarray. Click here for file

Additional file 12 **Module Membership and Binding Information in the Signed Zhou et al (2007) Network**. This file contains module membership, *k*_*ME*_, and binding data from Loh *et al *(2006), Boyer *et al *(2007), and Chen *et al *(2008) for each gene on the microarray. Click here for file
