# Supplementary material for: Signed weighted gene co-expression network analysis of transcriptional regulation in murine embryonic stem cells
Source: BMC Genomics. 2009 Jul 20;10:327. doi: 10.1186/1471-2164-10-327 (PMC2727539; doi:10.1186/1471-2164-10-327)

The Topological Overlap Map

(a) A representation of a simple network where the topological overlap map, $TOM(1,2) \approx a$ in figure. (b) A simple network where genes 1 and 2 are oppositely correlated with their neighbors resulting in differing topological overlap maps, $t_{1,2}$, when unsigned and signed similarity measures are employed.

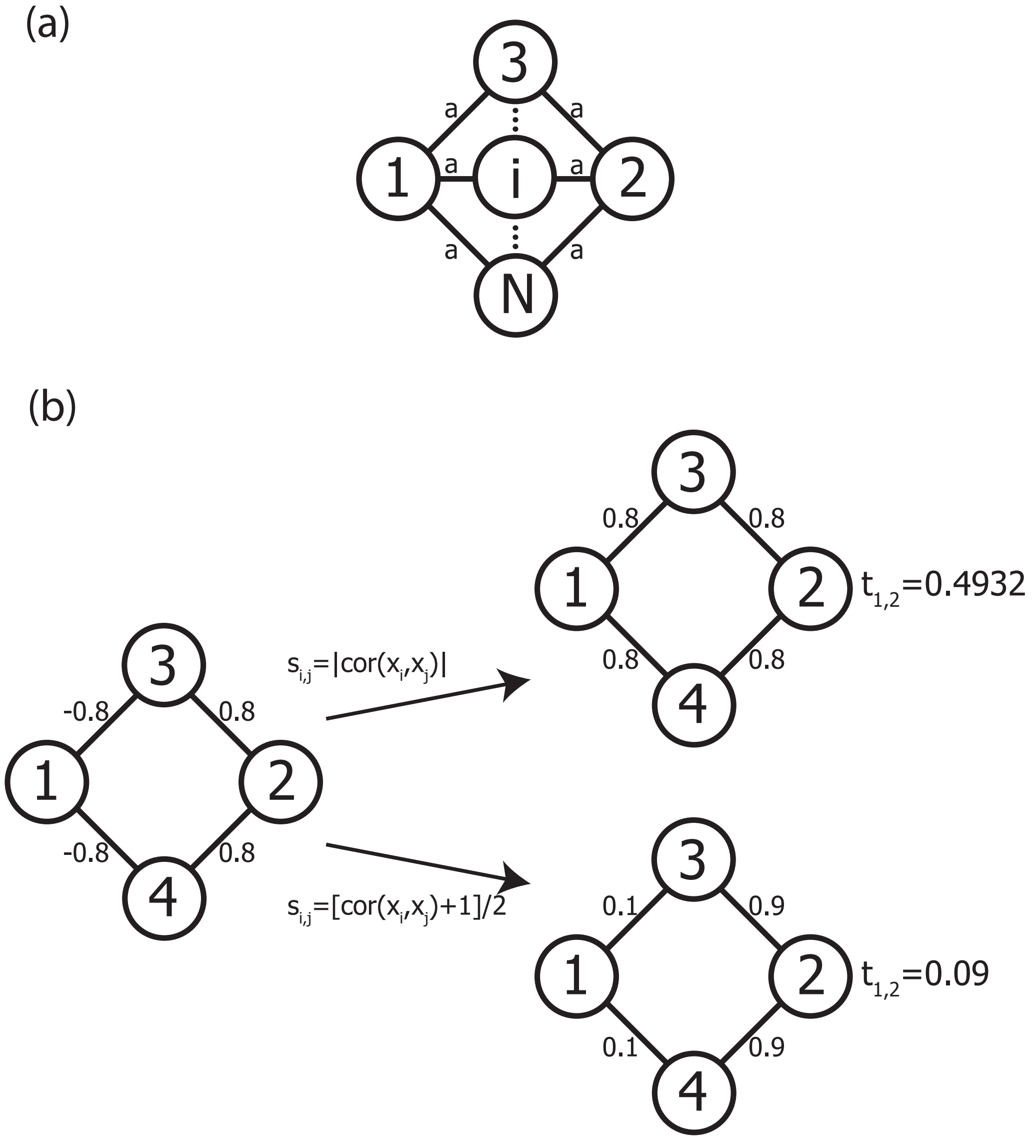

Supplement: Additional file 1 — A Simple Illustration of How the Choice of a Similarity Measure Affects TOM. The TOM measure of interconnectedness is often used to define clusters of highly interconnected genes. Here we use very simple networks to highlight properties of the TOM measure. (a) Computing the topological overlap between genes 1 and 2 when all connection strengths between intermediate genes equal the constant a. (b) The numbers on the edges of the left network are correlations while the numbers on the edges of the networks on the right hand side equal corresponding unsigned adjacencies (upper network) and signed adjacencies (lower network). In a signed network, the topological overlap between genes 1 and 2 is very low because intermediate genes 3 and 4 have negative correlations with gene 1. In contrast, the topological overlap between genes 1 and 2 is high in an unsigned network. [file 1471-2164-10-327-S1.pdf]
