# Supplementary material for: Signed weighted gene co-expression network analysis of transcriptional regulation in murine embryonic stem cells
Source: BMC Genomics. 2009 Jul 20;10:327. doi: 10.1186/1471-2164-10-327 (PMC2727539; doi:10.1186/1471-2164-10-327)

Intramodular and Module Eigengene Based Connectivity are Linear Related when Adjacency Parameter $\beta = 1$

Intramodular Connectivity $(k_i)^{\frac{1}{\beta}}$ (x-axis) is plotted versus Module Eigengene Base Connectivity k_{ME} (y-axis) for select modules from the signed network created from the Zhou *et al* data set (modules are colored accordingly).

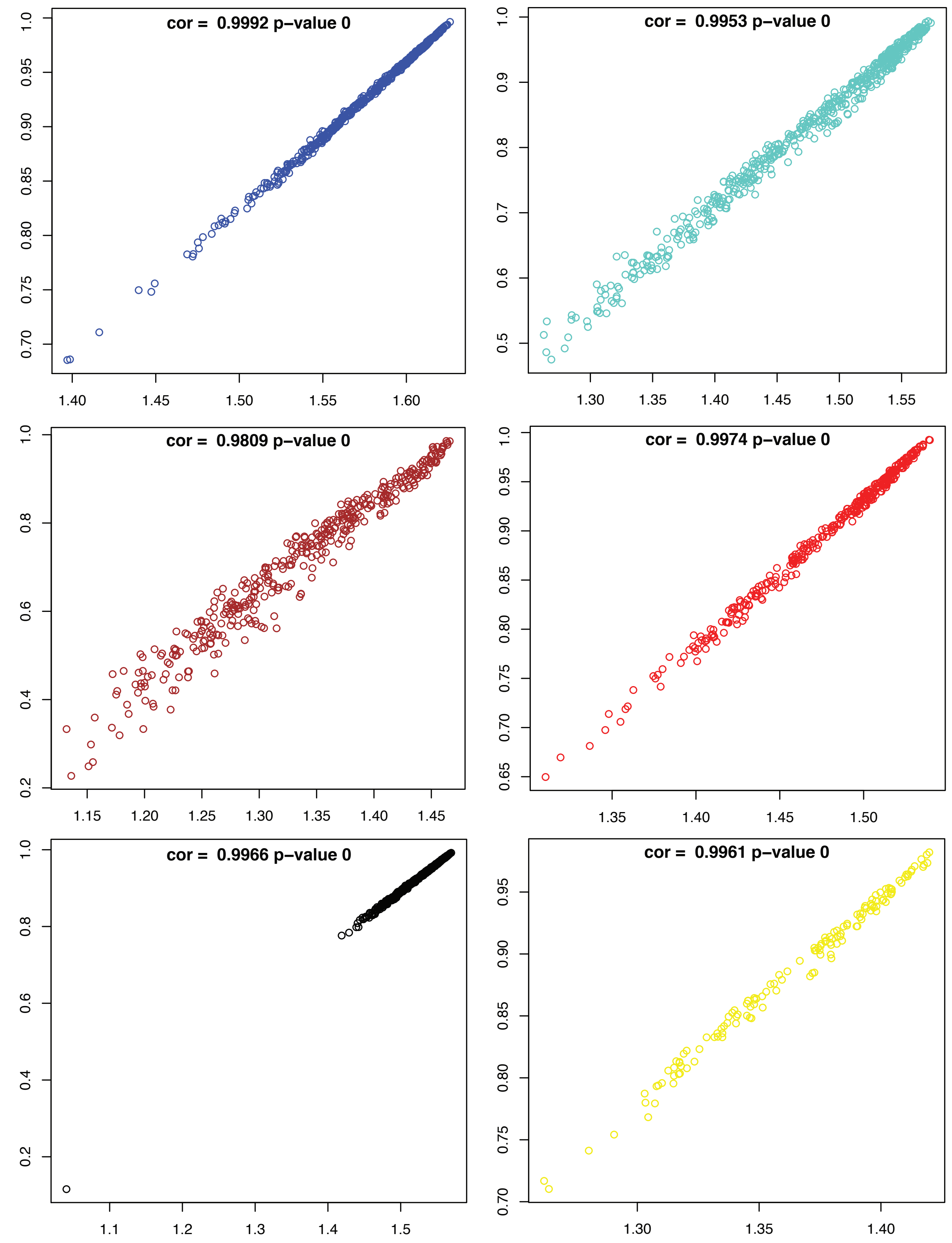

Supplement: Additional file 2 — Intramodular Connectivity is Highly Correlated with Module Eigengene Based Connectivity kME. For each module from the Zhou et al data, we plot intramodular connectivity (defined using a weighted network with power β = 1) versus module eigengene based connectivity kME. We find that the two connectivity measures are highly correlated. A theoretical derivation between network concepts and eigengene based analogs is presented in [32]. [file 1471-2164-10-327-S2.pdf]
