# Supplementary material for: Signed weighted gene co-expression network analysis of transcriptional regulation in murine embryonic stem cells
Source: BMC Genomics. 2009 Jul 20;10:327. doi: 10.1186/1471-2164-10-327 (PMC2727539; doi:10.1186/1471-2164-10-327)
Supplement: Additional file 4 — Comparing Gene Rankings to Regulators of Gene Expression. Here we relate different gene rankings to enrichment significance with regard to the following variables (a) histone H3K4 alone versus all others, (b) bivalent H3K4&H3K27 versus all others [49], (c) high CPG class versus all others (i.e. HCG versus ICG and LCG), (d) promoter CPG methylation status [50], (e) Oct 4 complex binding status, (f) cMyc complex binding status. We report results for 3 different gene rankings using the Ivanova data: the black and blue curve represent gene rankings according to and , respectively. The grey curve represents ranking according to a Student T-test of differential expression. Additional File 4 shows that black and blue module genes can have very different enrichment results that tend to be quite different from those of a standard analysis. [file 1471-2164-10-327-S4.pdf]

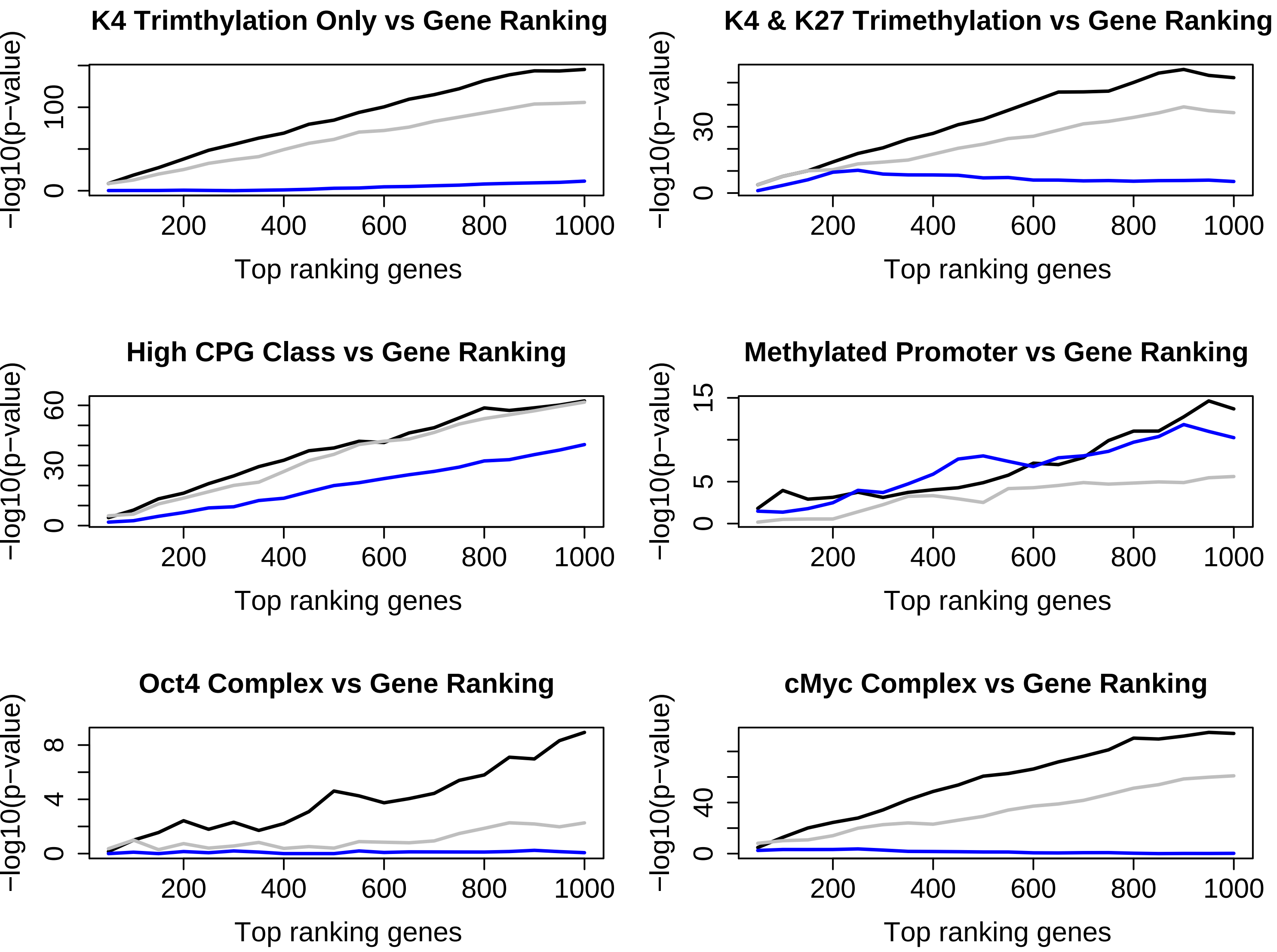
K4 Trimthylation Only vs Gene Ranking
−log10(p−value)
0
100
200
400
600
800
1000
Top ranking genes
K4 & K27 Trimethylation vs Gene Ranking
−log10(p−value)
0
30
200
400
600
800
1000
Top ranking genes
High CPG Class vs Gene Ranking
−log10(p−value)
0
30
60
200
400
600
800
1000
Top ranking genes
Methylated Promoter vs Gene Ranking
−log10(p−value)
0
5
15
200
400
600
800
1000
Top ranking genes
Oct4 Complex vs Gene Ranking
−log10(p−value)
0
4
8
200
400
600
800
1000
Top ranking genes
cMyc Complex vs Gene Ranking
−log10(p−value)
0
40
200
400
600
800
1000
Top ranking genes
