# Supplementary material for: Signed weighted gene co-expression network analysis of transcriptional regulation in murine embryonic stem cells
Source: BMC Genomics. 2009 Jul 20;10:327. doi: 10.1186/1471-2164-10-327 (PMC2727539; doi:10.1186/1471-2164-10-327)

Comparison of Functional Enrichment of Differentiation and Pluripotency Modules from Zhou *et al*

Ingenuity Pathway Analysis comparison of functional enrichments in the differentiation and pluripotency modules, colored blue and black, respectively. p-values have been corrected for multiple hypothesis test (Benjamini-Hochberg). Only significantly enriched classifications are shown.

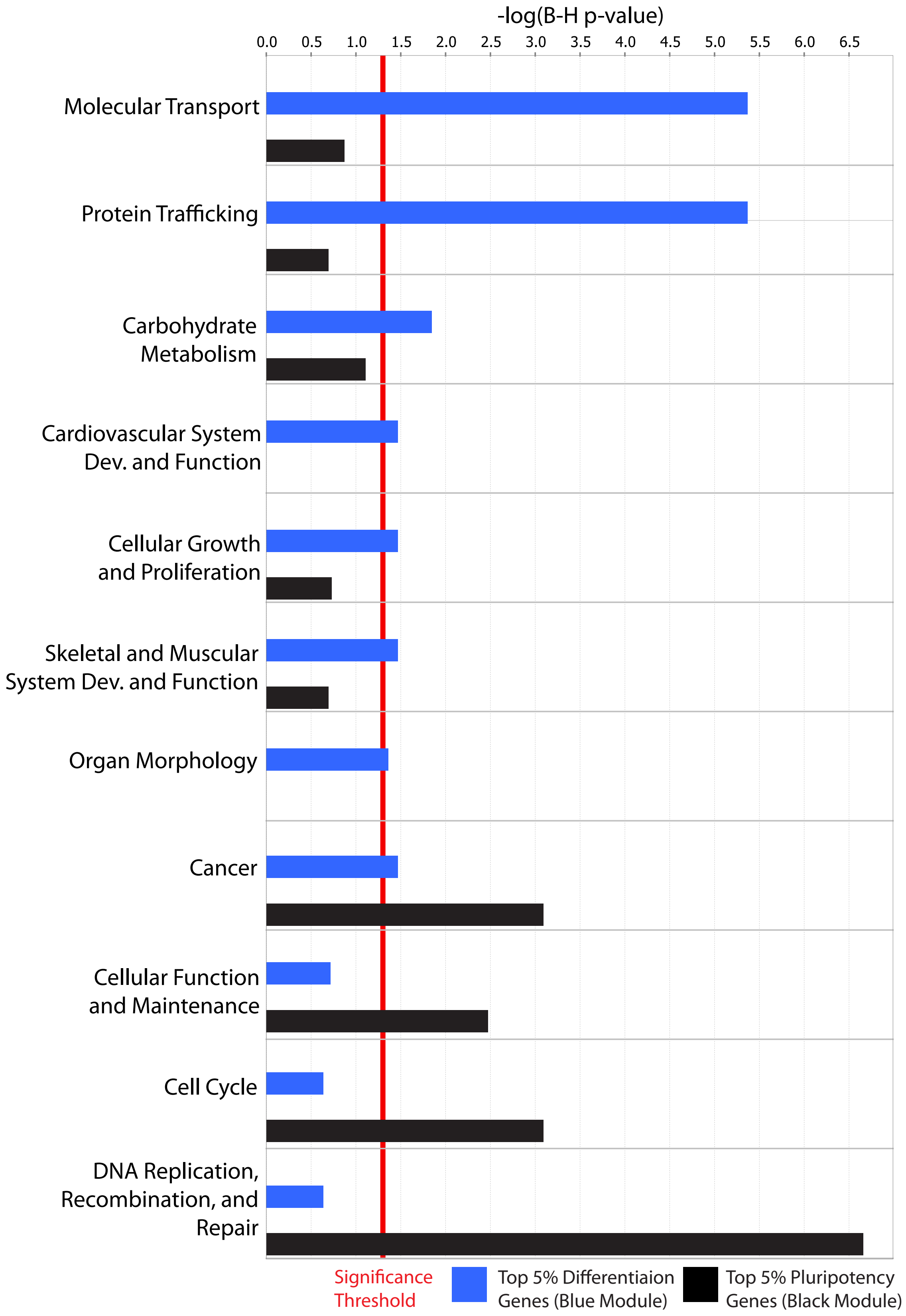

Supplement: Additional file 7 — Ingenuity Pathway Analysis of the Pluripotency and Differentiation Modules from Zhou et al (2007). [file 1471-2164-10-327-S7.pdf]
