# Supplementary material for: Signed weighted gene co-expression network analysis of transcriptional regulation in murine embryonic stem cells
Source: BMC Genomics. 2009 Jul 20;10:327. doi: 10.1186/1471-2164-10-327 (PMC2727539; doi:10.1186/1471-2164-10-327)

A Comparison of Overlap in the Zhou *et al* and Ivanova *et al* data sets

Upper Left: The overlap of 1000 genes most strongly down-regulated upon differentiation in both data sets , ranked Student's t-test. Lower Left: Overlap of 1000 genes most up regulated upon differentiation between both data sets, ranked Student's t-test. Upper Right: Overlap oft he 1000 genes most strongly connected to the pluripotency modules in both data sets. Lower Right: Overlap oft he 1000 genes most strongly connected to the differentiation modules in both data sets. p-values are computed using the hyper-geometric distribution.

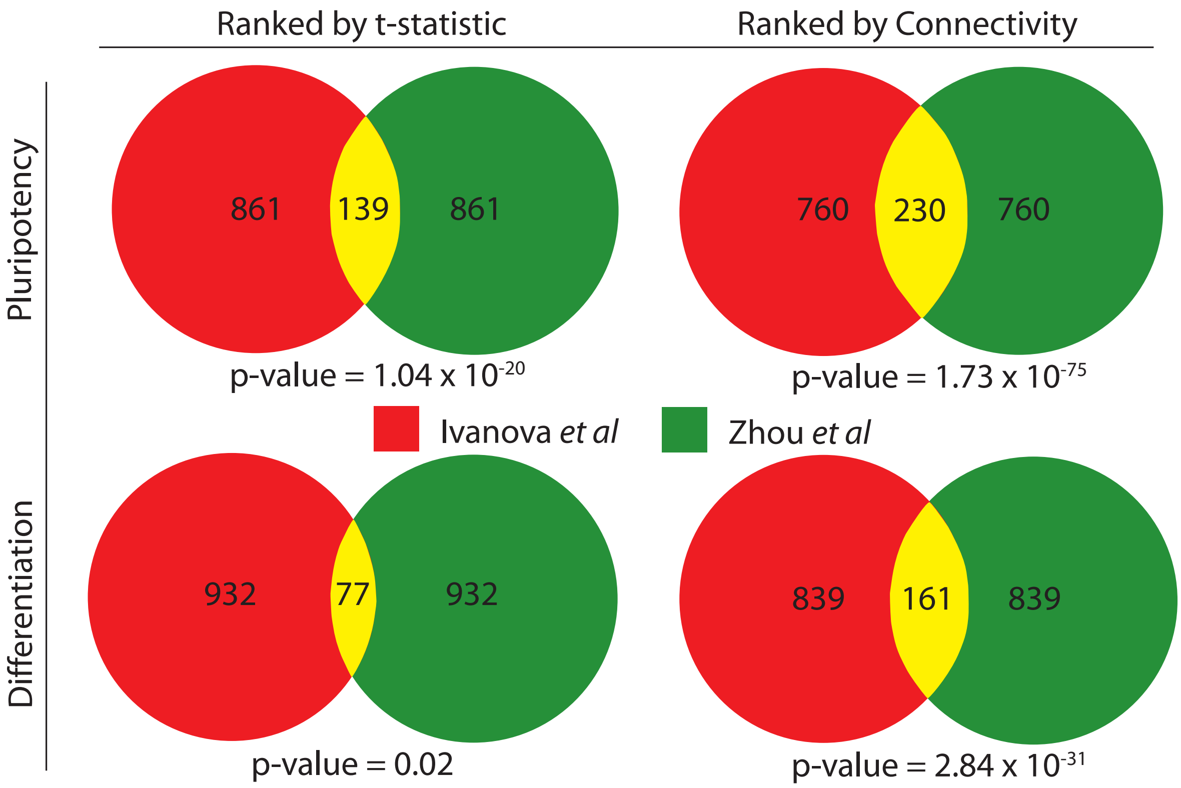

Supplement: Additional file 8 — Comparison of Overlap in Ivanova et al and Zhou et al (2007) when Ranking by t-statistic and Connectivity. [file 1471-2164-10-327-S8.pdf]
