# Supplementary material for: Signed weighted gene co-expression network analysis of transcriptional regulation in murine embryonic stem cells
Source: BMC Genomics. 2009 Jul 20;10:327. doi: 10.1186/1471-2164-10-327 (PMC2727539; doi:10.1186/1471-2164-10-327)

A Comparison of Genes Ranked by Network Connectivity and Differential Expression in the Zhou *et al* data set

Ingenuity Pathway Analysis off unctional enrichments in the set of genes ranked within the top 1000 by Student's t-test and k_{ME} and yet do not overlap with eachother. Venn diagrams show the amount of gene overlap in the top 1000 pluripotency genes and the 1000 genes most significantly down-regulated upon Oct4 RNAi (left) and gene overlap oft he top 1000 differentiation genes and the 1000 genes most significantly up-regulated with Oct4 RNAi (right). Significance of expression change was determined by Student's t-statistic. p-values have been corrected for multiple hypothesis test (Benjamini-Hochberg).

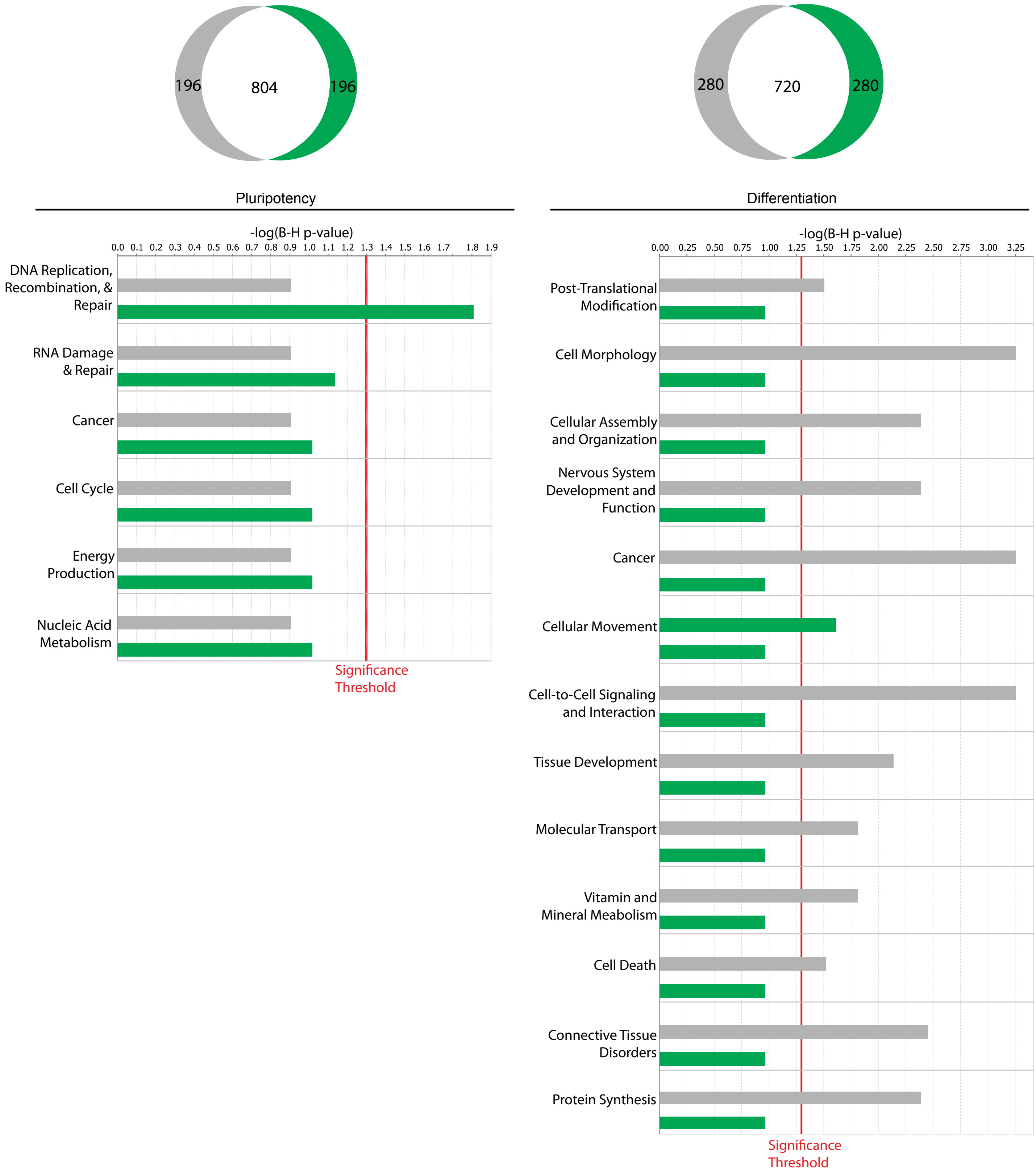

Supplement: Additional file 9 — Ingenuity Pathway Analysis of Genes Ranked by Connectivity and Differential Expression in Zhou et al (2007). [file 1471-2164-10-327-S9.pdf]
