# Supplementary figures and images for: Signed weighted gene co-expression network analysis of transcriptional regulation in murine embryonic stem cells
Source: BMC Genomics. 2009 Jul 20;10:327. doi: 10.1186/1471-2164-10-327 (PMC2727539; doi:10.1186/1471-2164-10-327)

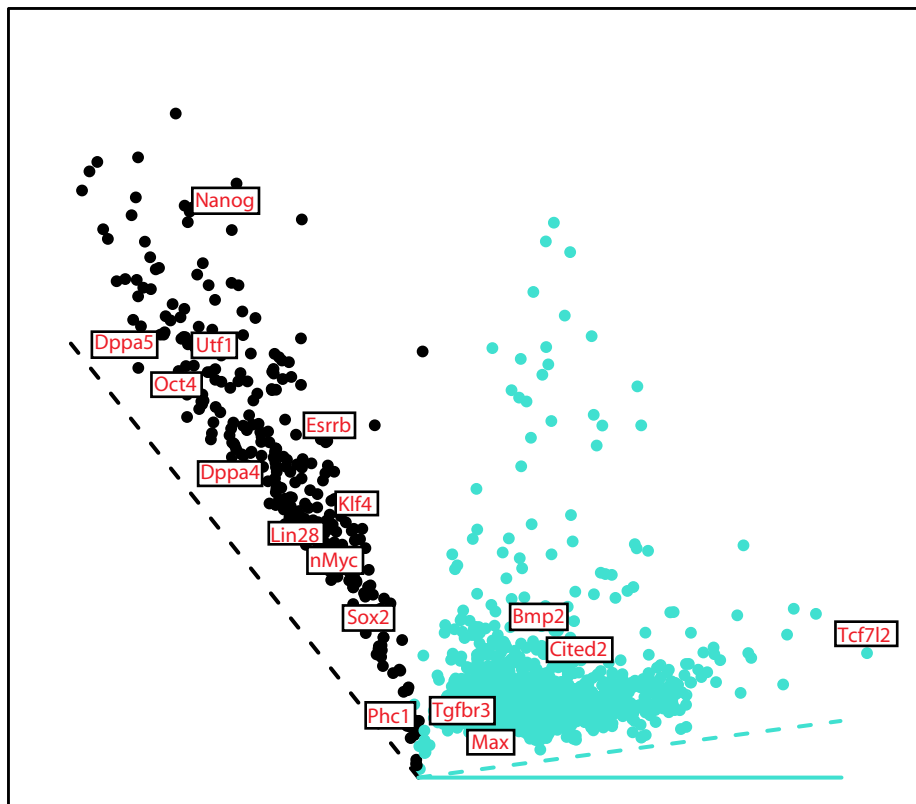

Supplement: Additional file 10 — Understanding Signed Module Membership. Here we visualize the relative position of unsigned and signed similarity modules. We used module eigengene based connectivity, kME = cor(xi, E), to visualize a gene's module membership, focusing on genes located in the signed turquoise or black modules. For any vectors a and b with angle θ between them, their correlation can be interpreted as cor(a, b) = cos(θ). Using this relationship we plotted genes in polar coordinates (radially) relative to the unsigned turquoise module. The figure shows the angle, θ, between the gene's expression profile and the turquoise module eigengene from the unsigned network, indicated by the solid turquoise line. Each gene's radius is defined as the absolute value of its log2 expression fold change (FC). FC is the ratio between the average expression in the control RNAi samples and the average expression in the Oct4 RNAi knock down samples. For reference the signed turquoise and black module eigengenes are indicated by dashed turquoise and black lines, respectively, and genes are colored by signed module membership. Known ES cell regulators and differentiation markers are labeled. Note that the signed module eigengenes (dashed lines) reflect the relationship within their corresponding modules while the unsigned module eigengene (solid line) reflects the relationship between the two signed modules. [file 1471-2164-10-327-S10.pdf]
